# Bone Marrow Mesenchymal Stem Cell‐Derived Dermcidin‐Containing Migrasomes enhance LC3‐Associated Phagocytosis of Pulmonary Macrophages and Protect against Post‐Stroke Pneumonia

**DOI:** 10.1002/advs.202206432

**Published:** 2023-05-28

**Authors:** Tiemei Li, Xiaotao Su, Pinglan Lu, Xinmei Kang, Mengyan Hu, Chunyi Li, Shisi Wang, Danli Lu, Shishi Shen, Huipeng Huang, Yuxin Liu, Xiaohui Deng, Wei Cai, Lei Wei, Zhengqi Lu

**Affiliations:** ^1^ Department of Neurology Mental and Neurological Disease Research Center the Third Affiliated Hospital of Sun Yat‐sen University Guangzhou 510630 China; ^2^ Center of Clinical Immunology Mental and Neurological Disease Research Center the Third Affiliated Hospital of Sun Yat‐sen University Guangzhou 510630 China; ^3^ Guangdong Provincial Key Laboratory of Brain Function and Disease Zhongshan School of Medicine Sun Yat‐sen University Guangzhou 510630 China; ^4^ Surgical Intensive Care Unit The Third Affiliated Hospital of Sun Yat‐sen University Guangzhou 510630 China

**Keywords:** bone marrow mesenchymal stem cell, macrophage, migrasome, post‐stroke pneumonia

## Abstract

Pneumonia is one of the leading causes of death in patients with acute ischemic stroke (AIS). Antibiotics fail to improve prognosis of patients with post‐stroke pneumonia, albeit suppressing infection, due to adverse impacts on the immune system. The current study reports that bone marrow mesenchymal stem cells (BM‐MSC) downregulate bacterial load in the lungs of stroke mice models. RNA‐sequencing of the lung from BM‐MSC‐treated stroke models indicates that BM‐MSC modulates pulmonary macrophage activities after cerebral ischemia. Mechanistically, BM‐MSC promotes the bacterial phagocytosis of pulmonary macrophages through releasing migrasomes, which are migration‐dependent extracellular vesicles. With liquid chromatography‐tandem mass spectrometry (LC‐MS/MS), the result shows that BM‐MSC are found to load the antibacterial peptide dermcidin (DCD) in migrasomes upon bacterial stimulation. Besides the antibiotic effect, DCD enhances LC3‐associated phagocytosis (LAP) of macrophages, facilitating their bacterial clearance. The data demonstrate that BM‐MSC is a promising therapeutic candidate against post‐stroke pneumonia, with dual functions of anti‐infection and immunol modulation, which is more than a match for antibiotics treatment.

## Graphical abstract

Pneumonia is one of the leading causes of death after cerebral ischemia. The current study demonstrates that bone marrow mesenchymal stem cells (BM‐MSCs) produce migrasomes containing dermcidin, which enhances LC3‐associated phagocytosis (LAP) of macrophages, and thus facilitates bacterial clearance. It is proposed that BM‐MSC is a promising therapy against post‐stroke pneumonia, with dual functions of anti‐infection and immunol modulation.

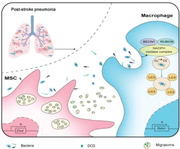

## 1 Introduction

As an immune‐privilege organ, damage of brain in acute ischemic stroke (AIS) exerts a powerful immunosuppressive effect that promotes fatal intercurrent infection.^[^ ^1^, ^2^ ^]^ Post‐stroke pneumonia is one of the leading causes of AIS patient's death.^[^ ^3^, ^4^ ^]^ Considering the detrimental impacts of post‐stroke pneumonia on stroke outcomes, antibiotics that restrict pathogen viability were once administered to stroke patients for infection prevention. Nevertheless, the strategy has soon been proved to be invalid for failing to improve stroke outcomes.^[^ ^5^ ^]^ One of the major adverse effects of antibiotics is the suppressing impact on macrophage activities.^[^ ^6^ ^]^ Phagocytosis and other bactericide functions of macrophages have been proved to be inhibited by several antibiotics, including ciprofloxacin, linezolid, etc.^[^ ^7^ ^]^

The immunological fine‐tuning functions of mesenchymal stem cells (MSC) have been widely reported.^[^ ^8^, ^9^ ^]^ Adoptive transfer of MSC ameliorates neural inflammation in stroke lesions,^[^ ^10^ ^]^ while enhancing macrophage/microglia phagocytosis of lesional debris.^[^ ^11^ ^]^ On the other hand, MSC treatment has been found to improve clinic outcomes of lung infection, including bacterial pneumonia caused by *E. coli* ^[^ ^12^ ^]^ and viral pneumonia including COVID‐19.^[^ ^13^ ^]^ Therefore, we hypothesize that MSC transfer could control the pathogen load in post‐stroke pneumonia and fill the gap of immunol inability in antibiotics treatment in the mean‐time.

The current study explores the therapeutic values of MSC transfer in post‐stroke pneumonia. We report that adoptive transfer of bone marrow mesenchymal stem cells (BM‐MSC) protects against post‐stroke pneumonia with beneficial stroke outcomes. Mechanistically, BM‐MSC promote the bacterial phagocytosis of pulmonary macrophages through releasing migrasomes, which are migration‐dependent extracellular vesicles. With liquid chromatography‐mass spectrometry (LC‐MS), we found that BM‐MSC‐derived migrasomes contain the antibacterial peptide dermcidin (DCD). DCD subsequently restricts bacteria growth and enhances LC3‐associated phagocytosis (LAP) of macrophages, which facilitates bacteria clearance and digestion. BM‐MSC transfer may represent a potential strategy for post‐stroke pneumonia prevention and treatment, which is more than a match for antibiotics.

## 2 Results

### 2.1 Bone Marrow Mesenchymal Stem Cells Transfer Protects against Acute Ischemic Stroke and Prevents Post‐Stroke Pneumonia

**Figure 1:**
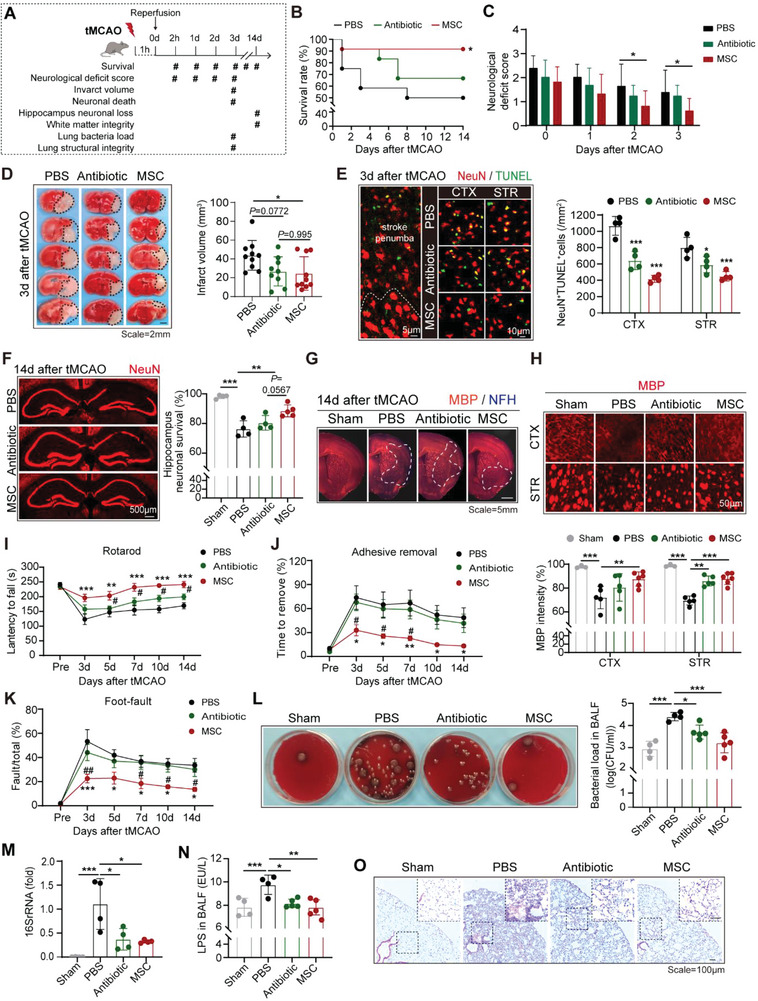
BM‐MSC transfer protects against AIS and prevents post‐stroke pneumonia. WT male C57/Bl6 mice were subjected to 60 min of tMCAO, then treated with BM‐MSC (2 × 10^6^ cells per mouse, i.v., single dose) at 2 h after reperfusion or broad‐spectrum antibiotic enrofloxacin (20 mg per kg per day, i.p) at 0–2d after stroke. Animals were sacrificed at 3d or 14d after tMCAO. A) Timeline of in vivo experimental design. B) Survival of mice in each group was recorded at 0–14 days after tMCAO. *n* = 12 in each group. ^*^ *p* < 0.05, compared with PBS‐treated group by log‐rank test. C) Neurological deficit score was assessed at 0–3d after tMCAO. *n* = 8 in PBS‐treated group, *n* = 9 in antibiotic‐treated group and *n* = 10 in BM‐MSC‐treated group. ^*^ *p* < 0.05, by two‐way ANOVA (mean ± standard deviation). D) Infarct volume of stroke mice was quantified with 2,3,5‐triphenyltetrazolium chloride (TTC) staining or immunostaining of NeuN with coronal sections collected at 3d after tMCAO. Representative images were displayed. Dashed lines outlined the infarct area. *n* = 9–10 in each group, ^*^ *p* < 0.05, compared with PBS‐treated group by one‐way ANOVA (mean ± standard deviation). E) Neuronal injury in stroke penumbra was evaluated with co‐immunostaining of NeuN (red) and TUNEL (green) at 3d after tMCAO. Representative images of NeuN^+^TUNEL^+^ neurons in stroke penumbra of cortex (CTX) and striatum (STR) were displayed. *n* = 4 in each group. ^*^ *p* < 0.05, ^***^ *p* < 0.001, compared with PBS‐treated group by one‐way ANOVA (mean ± standard deviation). Images with larger field of view have been displayed in Figure S1E (Supporting Information). F) Hippocampus neuronal loss at 14d after tMCAO was evaluated with NeuN (red) immunostaining. Representative images were displayed. *n* = 4 in Sham operated group, *n* = 4 in PBS‐ and Antibiotic‐treated group, *n* = 5 in BM‐MSC‐treated group. ^**^ *p* < 0.01, ^***^ *p* < 0.001; by one‐way ANOVA (mean ± standard deviation). G,H) White matter integrity at 14d after tMCAO was analyzed with MBP/NFH immunostaining. (G) Representative images of MBP/NFH immunostaining were displayed. The dash lines outlined the infarct area. *n* = 3 in Sham‐operated group, *n* = 5 in PBS‐ and Antibiotic‐treated groups, and *n* = 6 in BM‐MSC‐treated group. (H) Representative MBP immunostaining in the cortex (CTX) and striatum (STR) was displayed. Mean fluorescence intensity (MFI) of MBP in CTX and STR was calculated. Images with larger field of view have been shown in Figure S1F (Supporting Information). *n* = 3 in Sham operated group, *n* = 5 in PBS‐ and Antibiotic‐treated group, *n* = 6 in BM‐MSC‐treated group. ^**^ *p*<0.01, ^***^ *p* < 0.001, by one‐way ANOVA (mean ± standard deviation). I–K) Sensorimotor functions of the stroke models were evaluated by rotarod test (I), adhesive‐removal test (J), and foot‐fault test (K) at 3–14d after tMCAO. *n* = 8 in each group, ^*^ *p* < 0.05, ^**^ *p* < 0.01, ^***^ *p* < 0.001 in MSC‐ versus PBS‐treated group; ^#^ *p* < 0.05, ^##^ *p* < 0.01, in MSC‐ versus Antibiotic‐treated group, by two‐way ANOVA (mean ± standard error). L–O) Lung bacterial load and lung tissue integrity were assessed at 3d after tMCAO. (L) Quantification of bacterial load in lung was calculated by smearing the bronchoalveolar lavage fluid (BALF) in blood plates and culturing. Data were presented as colony‐forming units (CFU)/ml (log10). *n* = 4–5 in each group. ^*^ *p* < 0.05, ^***^ *p* < 0.001, by one‐way ANOVA (mean ± standard deviation). (M) Quantification of lung tissue 16S rRNA. *n* = 4 in each group. ^*^ *p* < 0.05, ^***^ *p* < 0.001, by one‐way ANOVA (mean ± standard deviation). (N) Lipopolysaccharide (LPS) level in BALF was assessed with ELISA. *n* = 4–5 in each group. ^*^ *p* < 0.05, ^**^ *p* < 0.01, by one‐way ANOVA (mean ± standard deviation). (O) Lung histopathology by hematoxylin and eosin (H&E) staining. *n* = 3 in each group. Representative images of each group were displayed.

Human BM‐MSC was successfully isolated. To identify BM‐MSC, the surface antigen profile and the differentiation ability of BM‐MSC were examined (Figure S1A,B, Supporting Information). To compare the therapeutic effects against AIS and post‐stroke pneumonia, wild‐type (WT) male C57/Bl6 mice were subjected to 60 min of transient middle cerebral artery occlusion (tMCAO) and then treated with BM‐MSC (2 × 10^6^ cells per mouse, i.v.) or a broad‐spectrum antibiotic Enrofloxacin (20 mg per kg per day, i.p.) at 2 h after reperfusion (**Figure** **1**A, Figure S1C,D, Supporting Information). In consistence with previous reports,^[^ ^14^ ^]^ BM‐MSC transfer increased the survival rate at 1–14 d after stroke (Figure 1B). The BM‐MSC recipients displayed reduced neurological deficit scores (Figure 1C) and diminished infarct volume (Figure 1D). Neuronal survival was preserved by BM‐MSC transfer at both 3d (short term, Figure 1E; Figure S1E, Supporting Information) and 14d (long term, Figure 1F) after stroke. Besides, BM‐MSC protected white matter integrity as revealed by MBP staining in both striatum (STR) and cortex (CTX) of the tMCAO models (Figure 1G,H; Figure S1F, Supporting Information). Accordingly, BM‐MSC‐treated stroke models showed improved sensorimotor functions as assessed with rotarod test (Figure 1I), adhesive‐removal test (Figure 1J) and foot‐fault test (Figure 1K) at 3–14 d after tMCAO. Nevertheless, mice treated with antibiotics did not show significant improvement in stroke outcomes compared with the vehicle (PBS)‐treated controls (Figure 1B–K). To analyze bacteria infection of the stroke mice, bronchoalveolar lavage fluid (BALF) was isolated and subjected to bacterial culture at 3d after tMCAO (Figure 1L). Lung infection was ubiquitously found in mice after tMCAO. Preventive antibiotic treatment limited bacterial growth in lung tissue (Figure 1L). Meanwhile, quantification of lung tissue 16S rRNA that non‐specifically detected the presence of bacteria (Figure 1M) and lipopolysaccharide (LPS) level in BALF (Figure 1N) indicated decrements of bacterial load in antibiotic‐treated mice (Figure 1L–N). Interestingly, BM‐MSC recipients displayed comparable limitations of bacterial infection with the antibiotic‐treated mice (Figure 1L–N). However, despite the anti‐bacteria effect of antibiotic, Enrofloxacin‐treated mice showed severe pulmonary inflammation as revealed by HE staining, which was similar to the PBS‐treated controls (Figure 1O). To be noticed, lungs of the BM‐MSC recipients displayed restricted immune cells infiltration and preserved lung tissue integrity (Figure 1O). The results demonstrated that BM‐MSC offered favorable dual protection against post‐stroke pneumonia, which restricted bacterial growth and promoted pulmonary inflammatory resolution.

### 2.2 BM‐MSC Transfer Protects against Post‐Stroke Pneumonia in Macrophage‐Dependent Manner

**Figure 2:**
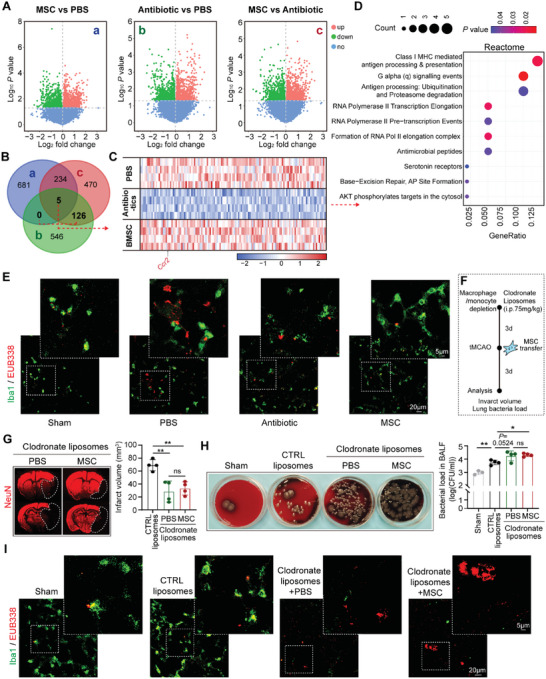
BM‐MSC transfer promotes bacterial clearance of macrophages. A–D) Lung tissue of stroke mice was isolated at 3d after tMCAO and subjected to bulk RNA sequencing (RNA‐seq). *n* = 4 in each group. (A) Differential expressed genes (DEGs) among PBS‐, antibiotic‐ and BM‐MSC‐treated groups. (B) Venn diagram showed the intersection of the up‐regulated DEGs in BM‐MSC‐ versus PBS‐treated groups (Aa), down‐regulated DEGs in antibiotic‐ versus PBS‐treated groups (Ab), and the up‐regulated DEGs in BM‐MSC‐ versus antibiotic‐treated groups (Ac). (C) Heatmap showing the DEGs in the intersection of PBS‐, antibiotic‐ and BM‐MSC‐treated groups. (D) Reactome pathway analysis of the DEGs in the intersection of PBS‐, antibiotic‐ and BM‐MSC‐treated groups. E) Mice were sacrificed at 3d after tMCAO. Lung sections were subjected to immunostaining of Iba1 (green) to label macrophages and colabeling with fluorescence in situ hybridization (FISH) of total bacteria (FISH probe EUB338, red). Experiments were repeated three times. F–I) Macrophages were depleted with clodronate liposomes (i.p., 75 mg kg^−1^). Administration of empty liposomes without clodronate was set as control (CTRL), and tMCAO was induced at 72 h after liposomes application. Mice were sacrificed at 3d after tMCAO. (F) Timeline of clodronate liposomes application and stroke outcome analysis. (G) Infarct volume of male mice was quantified with NeuN (red) immunostaining. Representative images were displayed. Dashed lines outlined the infarct area. *n* = 4 in each group, ^**^ *p* < 0.01, compared with CTRL liposomes group by one‐way ANOVA (mean ± standard deviation). (H) BALF was collected and cultured in blood plates. Data were expressed as CFU ml^−1^ (log10). *n* = 3–4 in each group. ^*^ *p* < 0.05, ^**^ *p* < 0.01, by one‐way ANOVA (mean ± standard deviation). (I) Lung sections were subjected to immunostaining of Iba1 (green) to label macrophages and FISH of total bacteria (FISH probe EUB338, red). Experiments were repeated three times.

Inflammatory cascades during post‐stroke pneumonia result in production of multiple inflammatory cytokines. The pulmonary cytokines could be rushed to the brain by blood flow which exacerbate neural inflammation.^[^ ^2^ ^]^ Therefore, timely resolution of pulmonary inflammation is necessity to stroke recovery.^[^ ^15^ ^]^ To obtain an overview of pulmonary inflammation of the BM‐MSC or Enrofloxacin treated mice, lung tissue of stroke models were isolated at 3d after tMCAO and subjected to bulk RNA sequencing (RNA‐seq). Differential expressed genes (DEGs) among PBS‐, antibiotic‐ and BM‐MSC‐treated stroke mice were analyzed (**Figure** **2**A). To explore the therapeutic mechanisms of BM‐MSC transfer, intersection of the up‐regulated DEGs in BM‐MSC‐ versus PBS‐treated mice (Figure 2Aa), down‐regulated DEGs in antibiotic‐ versus PBS‐treated mice (Figure 2Ab), and the up‐regulated DEGs in BM‐MSC‐ versus antibiotic‐treated mice (Figure 2Ac) were selected (Figure 2B). Signature genes of macrophage, including Ccr2, were found among the DEGs in the intersection (Figure 2C). DEGs in the intersection were further analyzed by Reactome pathway analysis. Results showed revealed that multiple antigen presentation‐associated pathways were up‐regulated in the lung of BM‐MSC‐treated mice (Figure 2D). Since macrophage is the most important bacterial scavenger in the lung,^[^ ^16^ ^]^ we reasoned that lung resident/recruited macrophage was implicated in BM‐MSC and offered protection against post‐stroke pneumonia.

To study the role of macrophage in the protection against post‐stroke pneumonia by BM‐MSC, lung sections of tMCAO models were subjected to immunol staining of Iba1 (macrophage marker, green) and colabeling with fluorescence in situ hybridization (FISH) of total bacteria (FISH probe EUB338, red). We found that BM‐MSC treatment enhanced bacterial clearance by macrophages as the bacteria were found to be largely retained within the Iba1^+^ cells (Figure 2E). In comparison, though the bacterial load was diminished in antibiotic‐treated mice, the residual bacteria remained to be unengulfed by macrophages, indicating that the anti‐bacterial function of macrophages was affected by the antibiotic treatment (Figure 2E). We further depleted the circulating monocytes (Figure 2F; Figure S2A,B, Supporting Information) and pulmonary resident macrophages (Figure 2F; Figure S2C,D, Supporting Information) with clodronate liposomes (75 mg kg^−1^, i.p., 3d before tMCAO) (Figure 2F). Strikingly, BM‐MSC failed to offer further protection against cerebral ischemia in monocyte/macrophage‐depleted mice (Figure 2G). Moreover, the anti‐bacterial effects of BM‐MSC were reversed (Figure 2H,I). Our data indicated functions of macrophages were indispensable in the protection against AIS and post‐stroke pneumonia by BM‐MSC transfer.

### 2.3 BM‐MSC Promotes Bacterial Clearance of Macrophages by Enhancing LC3‐Associated Phagocytosis

**Figure 3:**
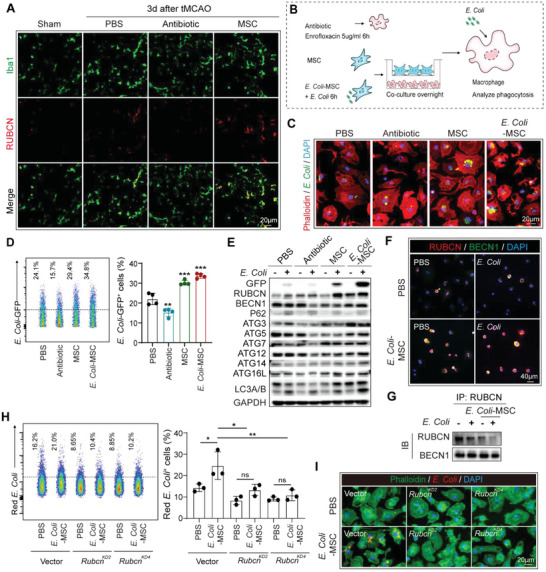
BM‐MSC promotes bacterial clearance of macrophages by enhancing LC3‐associated phagocytosis (LAP). A) Lung sections were subjected to immunostaining of Iba1 (green) to label macrophages and RUBCN (red) at 3d after tMCAO. Experiments were repeated three times. B–E) Analysis of phagocytic activity of bone marrow‐derived macrophages (BMDM). BMDM were treated with Enrofloxacin (5 µg ml^−1^, 6 h) or co‐cultured with BM‐MSC through a trans‐well overnight (pore diameter = 0.4 µm, BM‐MSC were stimulated with *E. Coli* for 6 h or not). BMDM were treated with GFP‐expressing *E. Coli* (*E. Coli*: BMDM = 20:1) for 1 h. A schematic diagram of in vitro experiment process was displayed (B). Phagocytic efficiency of BMDM was assessed with immunostaining (C) and flow cytometric analysis (D). Experiments were repeated four times. ^**^ *p* < 0.01, ^***^ *p* < 0.001, compared with PBS‐treated group by one‐way ANOVA (mean ± standard deviation). (E) BMDM were stimulated with or without *E. Coli* for 1 h. Expression of LAP and classic autophagic mediators were analyzed by western blot. Experiments were repeated three times. F) Immunofluorescence staining of BMDM with RUBCN (red) and BECN1 (green). G) Immunoprecipitation (IP) with anti‐RUBCN antibodies. IP product was analyzed with immunol blot (IB) to examine the interaction of RUBCN and BECN1. Experiments were repeated three times. H,I) Rubcn was knocked‐down in BMDM with Lentivirus‐shRNA (RUBCN^KD^). BMDM transfected with vector plasmid were set as control. Phagocytic efficiency of BMDM to *E. Coli* was assessed with flow cytometry (H) and immunostaining (I). Experiments were repeated three times. # ^*^ *p* < 0.05, ^**^ *p* < 0.01, by one‐way ANOVA (mean ± standard deviation).

We next explore the impacts of BM‐MSC on macrophages. The number of pulmonary macrophages after stroke was first quantified with flow cytometry, which revealed that the cell count was stable among PBS‐, antibiotic‐ and BM‐MSC treated mice (Figure S3A,B, Supporting Information). We next evaluated the functional alteration of macrophages after BM‐MSC treatment. As revealed by FISH of bacterial 16s rRNA, the efficiency of bacterial phagocytosis of pulmonary macrophages was improved in BM‐MSC transferred mice (Figure 2E). Invading bacteria are recognized and processed by macrophages through LC3‐associated phagocytosis (LAP).^[^ ^17^ ^]^ With immunol staining, we found that RUBCN (RUN domain and cysteine‐rich domain‐containing Beclin 1‐interacting protein), the specific marker of LAP, was upregulated in pulmonary macrophages of BM‐MSC recipients (**Figure** **3**A; Figure S4D, Supporting Information). Correspondingly, co‐culture of bone marrow‐derived macrophages (BMDM) with BM‐MSC through trans‐well (Figure 3B) improved their clearance of *E. Coli*, which is one of the major incumbent pathogens causing post‐stroke pneumonia (Figure 3C,D). With western blot, we found that BM‐MSC co‐culture upregulated RUBCN expression in BMDM and promoted the down‐stream LC3‐associated digestion of *E. Coli* (Figure 3D). Moreover, when BM‐MSC was stimulated with *E. Coli* (*E. Coli‐*BM‐MSC) to intimate the micro‐environment in post‐stroke pneumonia, the promoting effects of BMDM phagocytosis by BM‐MSC was further enhanced (Figure 3B–D). In human monocyte‐derived macrophages, we found that non‐stimulated BM‐MSCs failed to increase bacterial phagocytosis, while the *E. Coli*‐stimulated BM‐MSCs efficiently improve the bacterial clearance of macrophages (Figure S5A,B, Supporting Information). During LAP, RUBCN binds to BECN1, facilitating its assembly with PI3K complex, which subsequently activates NADPH oxidase (NOX), a necessary component of LAP.^[^ ^18^, ^19^ ^]^ We noticed that RUBCN displayed active assembly with BECN1 in mouse BMDM and human macrophages that co‐cultured with *E. Coli‐*BM‐MSC, indicating LAP progression (Figure 3E–G; Figure S5C–E, Supporting Information). When inhibiting LAP of BMDM by knocking down Rubcn (Figure S4H,I, Supporting Information), the promotion of BMDM bacterial clearing by *E. Coli‐*BM‐MSC was abolished (Figure 3H,I), which illustrated that BM‐MSC enhanced bactericidal function of macrophages through promoting LAP. To be noticed, Enrofloxacin seemed to downregulate bacterial clearance of BMDM and their expression of RUBCN (Figure 3C–E), which further demonstrated the superior protection against post‐stroke pneumonia of BM‐MSC compared with antibiotics.

### 2.4 BM‐MSC Promotes Bacterial Clearance by Macrophages through Releasing Migrasomes

**Figure 4:**
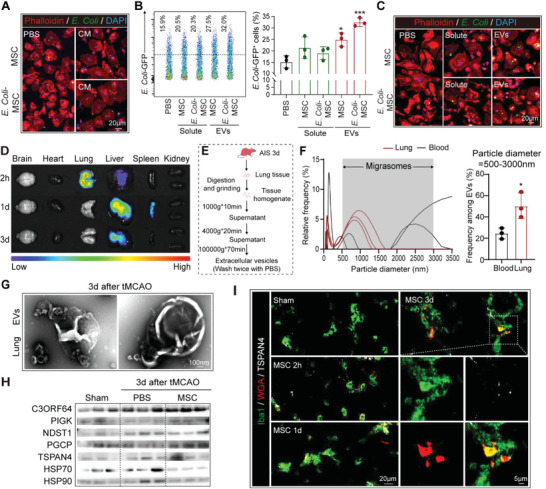
BM‐MSC transfer prevents post‐stroke pneumonia by releasing migrasomes. A–C) BMDM were treated with GFP expressing *E. Coli* (*E. Coli*: BMDM = 20:1), and the clearance effect was evaluated at 1 h. (A) BMDM were pre‐treated with the conditioned medium (CM) of BM‐MSC overnight. Phagocytic efficiency of BMDM was assessed with immunostaining. Experiments were repeated three times. (B,C) BMDM were pre‐treated with the solute or extracellular vesicles (EVs, 50 µg ml^−1^) of BM‐MSC overnight. Phagocytic efficiency of BMDM to *E. Coli*‐GFP was assessed with flow cytometry (B) and immunostaining (C). Experiments were repeated for three times. ^*^ *p* < 0.05, ^**^ *p* < 0.01, compared with PBS‐treated group by one‐way ANOVA (mean ± standard deviation). D) Ex vivo imaging of major organs from tMCAO mice at 2 h, 1d and 3d after intravenously injection of DiR‐labeled BM‐MSC (2 × 10^6^ cells per mouse). *n* = 3 in each group. E–H) EVs were isolated from lung tissue of mice at 3d after tMCAO. (E) Schematic diagram of the EVs separation process. (F) Particle diameter of EVs isolated from the lung or blood of BM‐MSC‐treated stroke mice were quantified. *n* = 3 in each group. ^*^ *p* < 0.05, compared with blood by Student's *t*‐test (mean ± standard deviation). (G) Transmission electron microscopy (TEM) analysis of the lung EVs isolated from BM‐MSC‐treated mice after negative staining. *n* = 3 in each group. (H) Expression of migrasome and exosome markers in lung EVs was analyzed with western blot. The experiments were repeated three times. I) Stroke mice were intravenously injected with WGA‐labeled BM‐MSC (red, 2×106 cells per mouse). Lung sections were collected at 2 h,1d and 3d after injection then subjected to immunostaining of Iba1 (green) to label macrophages and TSPAN4 (white) to label migrasome. *n* = 3 in each group. Representative images were displayed.

We further study the mechanism of how BM‐MSC improved bacterial clearance by macrophages. In the co‐culture system, BM‐MSC was separated from BMDM with an insert while their non‐contact communication was unaffected (Figure 3B). Conditioned medium (CM) of BM‐MSC or that pre‐treated with *E. Coli* could both enhance *E. Coli* (GFP^+^) phagocytosis by BMDM (**Figure** **4**A), indicating that it was BM‐MSC‐derived secretive factors that promoted BMDM phagocytosis of bacteria. BM‐MSC‐derived CM was further separated into soluble fraction and extracellular vesicles (EVs). We recorded that EVs, especially those produced by *E. Coli* pre‐treated BM‐MSC, enhanced bacterial engulfment by BMDM (Figure 4B,C). As for in vivo, BM‐MSC only displayed a short stay in the lung for ∼24 h after injection (Figure 4C). BM‐MSC seemed to retract from the lung within 2d while the anti‐bacterial effects of BM‐MSC transfer were still evident for at least 3d (Figure 1I–K). Therefore, we reasoned that it was BM‐MSC‐derived EVs that exerted pro‐phagocytosis effects on macrophages. We thus sought to identify the functional EVs in lungs of BM‐MSC‐transferred mice. Enrichment of EVs with a diameter of 500–3000 nm was found in the lung of BM‐MSC recipients, which coincided with the reported size of the newly discovered EV of migrasome (Figure 4E,F).^[^ ^20^ ^]^ Transmission electron microscopy (TEM) analysis of the EVs isolated from BM‐MSC CM and BM‐MSC transferred stroke models revealed typical migrasome morphology which was large vesicle binding to retraction fibers and containing small vesicles (Figure 4G,H). Moreover, western blot analysis of the lung tissue from BM‐MSC transferred stroke mice (3d after tMCAO) displayed increased expression of migrasome markers of C3ORF64, PIGK, and PGCP, while the level of exosome markers including HSP70 and HSP90 was stable comparing with the PBS‐injected controls (Figure 4H). Consistently, immunostaining demonstrated the retention of BM‐MSC (WGA pre‐stained, red) derived migrasomes (TSPAN4^+^, white) in the lung of tMCAO recipients at 0–3d after injection (Figure 4I).

**Figure 5:**
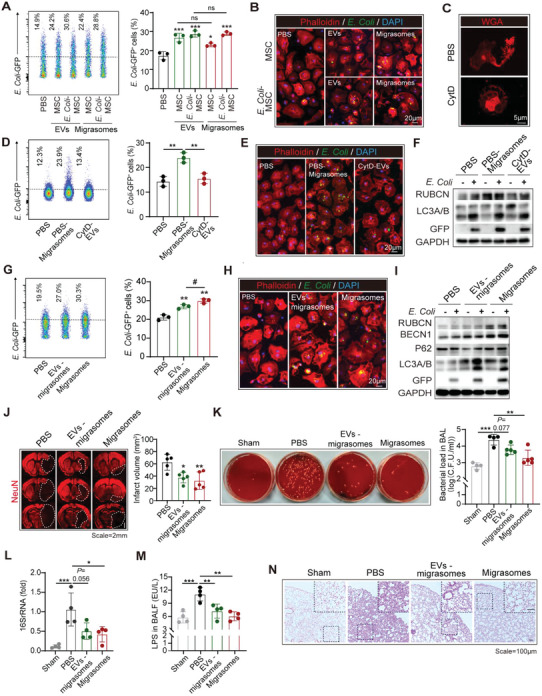
BM‐MSC promotes bacterial clearance by macrophages through releasing migrasomes. A,B) BMDM were pre‐treated with EVs (50 µg ml^−1^) or migrasomes (50 µg ml^−1^) that were derived from BM‐MSC overnight. GFP‐expressing *E. Coli* was treated to BMDM for 1 h (*E. Coli* : BMDM = 20:1). Phagocytic efficiency was assessed with flow cytometry (A) and immunostaining (B). Experiments were repeated three times. ^*^ *p* < 0.05, ^***^ *p* < 0.001, compared with PBS‐treated group by one‐way ANOVA (mean ± standard deviation). C–E) Cytochalasin D (CytD) was applied to inhibit cell migration (10 µm, 6 h). (C) Morphology of BM‐MSC was outlined with WGA (red). Phagocytic efficiency of BMDM to GFP expressing *E. Coli* (*E. Coli* : BMDM = 20:1, 1 h) was assessed with and flow cytometry (D) and immunostaining (E). Experiments were repeated three times. ^**^ *p* < 0.01, by one‐way ANOVA (mean ± standard deviation). F) Expression of LAP mediators was analyzed by western blot. Experiments were repeated three times. G–I) BMDM were pre‐treated with EVs – migrasoems (50 µg ml^−1^) or migrasomes (50 µg ml^−1^) that derived from BM‐MSC overnight. Phagocytic efficiency of BMDM to GFP expressing *E. Coli* (*E. Coli* : BMDM = 20 : 1, 1 h) was assessed with flow cytometry (G) and immunostaining (H). Experiments were repeated for three times. ^**^ *p* < 0.01, compared with PBS group by Student's *t*‐test (mean ± standard deviation). ^#^ *p* < 0.05, EVs – Migrasomes‐treated group compared with Migrasomes‐treated group by Student's *t*‐test (mean ± standard deviation). (I) Expression of LAP mediators was analyzed by western blot. Experiments were repeated three times. J–N) WT male C57/Bl6 mice were subjected to 60 min of tMCAO, then treated with EVs ‐migrasomes or migrasomes (10 mg kg^−1^, i.v.) at 2 h after reperfusion. Animals were sacrificed at 3d after tMCAO. (J) Infarct volume of male mice was quantified with immunostaining of NeuN (red). Representative images were displayed. Dashed lines outlined the infarct area. *n* = 5 in each group, ^**^ *p* < 0.01, by one‐way ANOVA (mean ± standard deviation). (K) BALF was collected and cultured in blood agar plates. Data were expressed as CFU ml^−1^ (log10). *n* = 4–5 in each group. ^**^ *p* < 0.01, ^***^ *p* < 0.001, by one‐way ANOVA (mean ± standard deviation). (L) Quantification of 16S rRNA in lunge tissue. *n* = 4 in each group. ^*^ *p* < 0.05, ^***^ *p* < 0.001, by one‐way ANOVA (mean ± standard deviation). (M) LPS level in BALF was assessed with ELISA. *n* = 4 in each group. ^*^ *p* < 0.05, ^**^ *p* < 0.01, ^***^ *p* < 0.001, by one‐way ANOVA (mean ± standard deviation). (N) Lung histopathology by H&E staining. *n* = 3 in each group. Representative images of each group were displayed.

We thus went on to investigate the pro‐phagocytic effects of BM‐MSC‐derived migrasomes. We recorded that BM‐MSC produced migrasomes in fibronectin‐coated plate (PBS treated, designated as PBS‐migrasome, PBS‐M) and the migrasome production was upregulated upon *E. Coli* stimulation (designated as *E. Coli*‐migrasome, *E. Coli*‐M) (Figure S4A–C, Supporting Information). As assessed with flow cytometric (**Figure** **5**A) and immunol staining (Figure 5B), we found that both PBS‐M and *E. Coli*‐M up‐regulated BMDM phagocytosis of GFP^+^ *E. Coli*. In the mean‐time, expression of RUBCN in BMDM was upregulated by PBS‐M or *E. Coli*‐M (Figure S7H, Supporting Information). To explore the necessity of migrasome in the enhancement of BMDM bacterial clearance offered by BM‐MSC, cytochalasin‐D (CytD, 10 µm) was applied to BM‐MSC to inhibit cellular migration(Figure 5C). EVs derived from CytD‐treated BM‐MSC (CytD‐EV), which did not contain migrasomes, were then treated to BMDM. We found that CytD‐EV could neither enhance GFP^+^ *E. Coli* phagocytosis by BMDM (Figure 5D–F), nor upregulate their expression of RUBCN (Figure 5F). On the other hand, the pro‐phagocytic effect of BM‐MSC‐derived migrasomes was comparable to that of BM‐MSC‐derived EVs (Figure 5A,B; Figure S7H, Supporting Information). In previous reports that investigated the therapeutic effects of BM‐MSC‐derived EVs, some of the studies utilized EVs that contained migrasomes,^[^ ^21^, ^22^ ^]^ while others excluded migrasomes in the EV formulation.^[^ ^23^ ^]^ In the current study, we found that macrophages treated with migrasomes displayed similar improvement in bacterial clearance as those treated with migrasome‐containing total EVs (Figure 5A,B), while those treated with migrasome‐excluded EVs showed inferior phagocytic enhancement (Figure 5G–I). In animal experiments, we found that either migrasomes or migrasome‐excluded EVs effectively diminished the infarct volume (Figure 5J). However, in in vivo analysis of post‐stroke lung infection, we found that tMCAO models transferred with migrasomes had decrement of bacterial growth in lung tissue (Figure 5K), lung tissue 16S rRNA concentration (Figure 5L), LPS level in BALF (Figure 5M) and pulmonary inflammation (Figure 5N), which was similar to those received BM‐MSC treatment (Figure 1L–O) and was superior to those transferred with migrasome‐excluded EVs (Figure 5K–N). Our data suggest that, in regard to post‐stroke pneumonia, we could expect more favorable therapeutic effects if migrasomes would be included in the EV formulation delivered to animals. Interestingly, BM‐MSC and their migrasome products also exhibited bactericidal function. *E. Coli* that co‐cultured with BM‐MSC or migrasomes displayed growth restriction (Figure S7I, Supporting Information). Therefore, we concluded that BM‐MSC promoted bacterial clearance by macrophages through releasing migrasomes and exerted bactericidal effects at the mean time.

### 2.5 Dermcidin (DCD) is Packed in BM‐MSC‐Derived Migrasomes and Improves Bacterial Clearance of Macrophages

**Figure 6:**
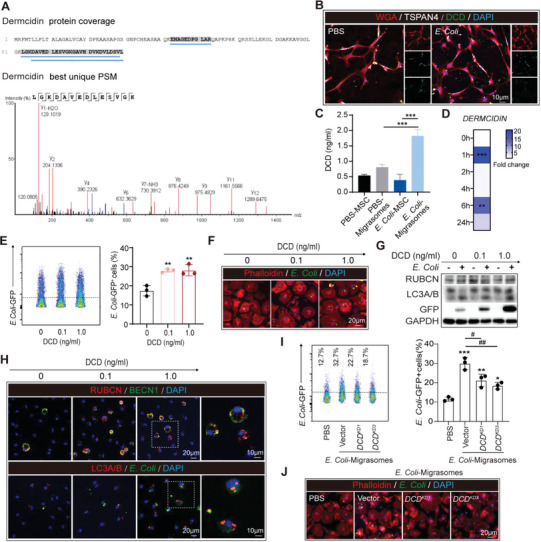
BM‐MSC‐derived migrasome contains dermcidin (DCD) and improves bacterial clearance of macrophages. A) PBS‐migrasomes were isolated and subjected to liquid chromatography‐mass spectrometry (LC‐MS) analysis. Protein coverage and best peptide‐spectrum (PSM) of PBS‐M were displayed. B,C) BM‐MSC were stimulated with *E. Coli* (*E. Coli* : MSC = 20:1, 6 h). (B) Immunostaining was used to evaluate the expression and localization of dermcidin (DCD, green) in BM‐MSC. Cell membrane was labeled with WGA (red) and migrasomes were labeled with TSPAN4 (white). (C) DCD level in BM‐MSC or migrasomes derived from PBS‐ or *E. Coli*‐treated BM‐MSC was assessed with ELISA. Experiments were repeated three times. ^***^ *p* < 0.001, by one‐way ANOVA (mean ± standard deviation). D) The mRNA level of DCD in BM‐MSC was assessed with QPCR at 0–24 h after *E. Coli* stimulation (*E. Coli* : MSC = 20:1). ^**^ *p* < 0.01, ^***^ *p* < 0.001, compared with 0 h group by one‐way ANOVA (mean ± standard deviation). E–H) BMDM were first pre‐treated with DCD (0–1 ng ml^−1^, overnight), and treated with *E. Coli* (*E. Coli*: BMDM = 20:1, 1 h). Phagocytic efficiency of BMDM to *E. Coli*‐GFP was assessed with flow cytometry (E) and immunostaining (F). The experiment was repeated three times. ^**^ *p* < 0.01, compared with DCD (0 ng ml^−1^) group by one‐way ANOVA (mean ± standard deviation). (G) Expression of LAP mediators was analyzed by western blot. Experiments were repeated three times. (H) Immunofluorescence staining showed the localization of LAP mediators in BMDM. I,J) DCD knock‐down (DCD^KD^) BM‐MSC was constructed by transfection of Lentivirus carrying DCD‐shRNA. DCD^KD^ or vector‐transfected BM‐MSC were stimulated with *E. Coli*. Migrasomes derived from BM‐MSC were isolated and treated to BMDM (50 µg ml^−1^ overnight). Phagocytic efficiency of BMDM to GFP expressing *E. Coli* (*E. Coli* : BMDM = 20:1, 1 h) was assessed with flow cytometry (I) and immunostaining (J). Experimental process was schematically displayed in Figure S7G (Supporting Information). Experiments were repeated three times. ^#^ *p* < 0.05, ^##^ *p* < 0.01, compared with BMDM treated with vector‐transfected *E. Coli*‐stimulated BM‐MSC derived migrasomes by one‐way ANOVA (mean ± standard deviation), ^*^ *p* < 0.05, ^**^ *p* < 0.01, ^***^ *p* < 0.001, compared with PBS‐treated BMDM group by one‐way ANOVA (mean ± standard deviation).

To explore the responsible molecule for the antibacterial effects of BM‐MSC‐derived migrasomes, PBS‐M was isolated and subjected to liquid chromatography tandem mass spectrometry (LC‐MS/MS) analysis. Among the identified components, the antimicrobial protein dermcidin (DCD) was notable (**Figure** **6**A). With immunostaining (Figure 6B) and ELISA (Figure 6C), we validated that DCD was concentrated in BM‐MSC‐derived migrasomes. The concentration of DCD was much lower in the cell body of BM‐MSC (Figure 6B,C). Moreover, as assessed with ELISA (Figure 6C) and QPCR (Figure 6D), we found that *E. Coli* stimulation upregulated DCD expression in BM‐MSC, which explained the super active effects of *E. Coli* pre‐treated BM‐MSC co‐culture and *E. Coli*‐M on macrophages.

We next evaluated the impacts of DCD on macrophage phagocytosis. To rule out bactericidal effect of DCD, BMDM were first pre‐treated with DCD (1 ng ml^−1^, overnight) before being subjected to bacterial clearing experiments. With immunostaining, we showed that DCD‐priming improved the *E. Coli* engulfment or myelin debris clearance by BMDM in dose‐dependent manner (Figure 6E,F; Figure S8B, Supporting Information), which revealed the pro‐scavenging function of DCD. In the meantime, DCD promoted LAP of macrophages after *E. Coli* engulfment (Figure 6G). As assessed with immunol staining, we found that co‐localization of RUBCN and BECN1, GFP^+^ *E. Coli* or and LC3A/B was upregulated in DCD pre‐treated BMDM (Figure 6H). To be noticed, the pro‐LAP function of DCD^KD^ BM‐MSC‐derived migrasomes (Figure S8E–G, Supporting Information) was abolished, which demonstrated the indispensable role of DCD in the antibacterial effects of BM‐MSC‐derived migrasomes (Figure 6I,J; Figure S8H, Supporting Information).

**Figure 7:**
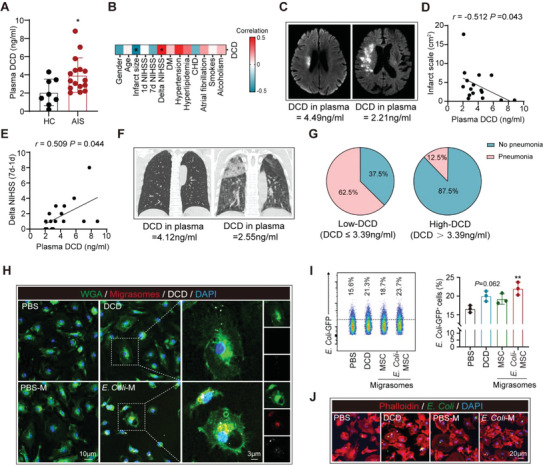
DCD is beneficial to AIS recovery and DCD‐containing BM‐MSC‐derived migrasome effectively promotes phagocytosis of macrophages. A–G) Peripheral blood of AIS patients (acute phase, 0–3d after disease onset, *n* = 16) and healthy controls (HC, *n* = 8) were collected. (A) Plasma DCD concentration was assessed with ELISA. ^*^ *p* < 0.05, compared with HC by Student's *t*‐test (mean ± standard deviation). (B) Correlation of clinic parameters and plasma DCD concentration was assessed with Spearman correlation analysis and Point‐biserial correlations. ^*^ *p* < 0.05. DM, diabetes mellitus, CHD, coronary heart disease. (C) Representative images of the magnetic resonance diffusion weighted imaging (MR‐DWI) of AIS patients with low plasma DCD concentration (DCD ≤ 3.33 ng ml^−1^) or high plasma DCD concentration (DCD > 3.33 ng ml^−1^). (D) Association between plasma DCD concentration with infarct scale was estimated with Spearman correlation analysis. (E) Association between plasma DCD concentration with delta NIHSS (NIHSS at 7d minus NIHSS at 1d) was estimated with Spearman correlation analysis. (F) Representative images of the chest Computed Tomography (CT) of AIS patients with low plasma DCD concentration (DCD ≤ 3.39 ng ml^−1^, median of the cohort) or high plasma DCD concentration (DCD > 3.39 ng ml^−1^, median of the cohort). (G) Pie charts showing the occurrence of post‐stroke pneumonia in AIS patients with low and high plasma DCD concentrations. H) DCD (1 ng ml^−1^), PBS‐migrasomes (PBS‐M, 50 µg ml^−1^) or *E. Coli*‐migrasomes (*E. Coli*‐M, 50 µg ml^−1^) labeled with Dil (red) were treated to BMDM (15 min). Immunostaining of WGA (green) and DCD (withe) in migrasome‐treated BMDM was performed. Experiments were repeated for three times. I,J) BMDM were first pre‐stimulated with DCD (1 ng ml^−1^), PBS‐M (50 µg ml^−1^), or *E. Coli*‐M (50 µg ml^−1^) for overnight then treated with *E. Coli* (*E. Coli* : BMDM = 20:1, 1 h). Phagocytic efficiency of BMDM to GFP expressing *E. Coli* was assessed with flow cytometry (I) and immunostaining (J). Experiments were repeated three times. ^**^ *p* < 0.01, compared with PBS‐treated group by one‐way ANOVA (mean ± standard deviation).

We further study the therapeutic potential of DCD and DCD‐containing BM‐MSC‐derived migrasomes. We found that DCD concentration in the peripheral blood was upregulated in AIS patients (acuter phase, 0–3d after disease onset) compared with age and gender‐matched healthy controls (HC) (**Figure** **7**A). Interestingly, DCD level in peripheral blood was negatively correlated with infarct volume in patients (Figure 7B–D), and positively correlated with delta NIHSS (7d–1d) (Figure 7B,E), illustrating that DCD was beneficial to AIS recovery. We further compared the post‐stroke pneumonia conditions in stroke patients with different plasma DCD levels. According to chest Computed Tomography (CT) or X‐ray analysis, we found that AIS patients with pneumonia displayed decreased plasma DCD concentration than those without (Figure S9A, Supporting Information). On the other hand, AIS patients with high plasma DCD concentration (DCD > 3.39 ng ml^−1^, median of the cohort) had more pronounced occurrence of pneumonia than those with low DCD level (DCD ≤ 3.39 ng ml^−1^, median of the cohort) (Figure 7F,G). However, the inflammation indicators of procalcitonin (PCT, Figure S9B, Supporting Information), CRP (Figure S9C, Supporting Information) and IL‐6 (Figure S9D, Supporting Information) were comparable between patients with high or low plasma DCD. Considering the anti‐bacterial effects of DCD^[^ ^24^ ^]^ and DCD‐containing macrophage‐derived migrasomes (Figure 7H–J), we infer that DCD is critical for the anti‐bacteria reaction against pneumonia.

Nevertheless, although direct DCD treatment was able to enhance macrophage phagocytosis, therapeutic effects of DCD‐containing BM‐MSC‐derived migrasomes seemed to be more effective. When treated with BMDM, DCD, and DCD‐containing BM‐MSC‐derived migrasomes were both engulfed by BMDM (Figure 7H). With flow cytometric analysis (Figure 7I) and immunol staining (Figure 7J), we found that BMDM treated with BM‐MSC‐derived migrasomes (50 µg ml^−1^.overnight) displayed more potent bacterial clearance than those treated with DCD protein (1 ng ml^−1^.overnight). Nevertheless, we failed to document immunomodulatory effects of DCD on BM‐MSC, which is established in BM‐MSC (Figure S8D, Supporting Information). Therefore, DCD‐containing BM‐MSC‐derived migrasome is a eutherapeutic therapy against post‐stroke pneumonia, which is superior to DCD protein as far as we are concerned.

## 3 Discussion

The current study reveals that BM‐MSC protects against post‐stroke pneumonia. BM‐MSC‐derived migrasomes, which are packed with DCD, display dual effects that reduce pulmonary bacteria load and enhance LAP of macrophages. We propose that BM‐MSC as well as the anti‐bacterial migrasomes are promising therapy against post‐stroke pneumonia.

Considering the life‐threatening impacts of post‐stroke pneumonia, treatment of lung infection prevention is of necessity. Antibiotics have been considered as a preventive treatment against post‐stroke pneumonia. In agreement with previous reports,^[^ ^25^, ^26^ ^]^ we recorded that antibiotic treatment exerted certain protection in animal models. Antibiotic‐treated mice displayed decreased infarct volume when compared with non‐treated mice. The therapeutic effect could be attributed to the inhibition of the detrimental impacts of post‐stroke infection. However, as assessed with the rotarod, adhesive removal and foot‐fault tests, antibiotic‐treated tMCAO models did not show improvement in sensorimotor functions, which coincided with the ineffectiveness of antibiotics in improving long‐term stroke outcomes.^[^ ^27^, ^28^ ^]^ Besides, the adverse effects of antibiotics on macrophage phagocytosis were evident, which further exacerbated the disorder of microbiota caused by antibiotics.^[^ ^29^, ^30^ ^]^ In comparison, BM‐MSC transfer offered multiple protection against tMCAO. BM‐MSC‐treated mice displayed smaller infarct volume, improved survival, and favorable neurological functions. To our interest, the protective efficacy against post‐stroke pneumonia of BM‐MSC was not inferior to antibiotics. Mechanistically, the bactericidal effects of BM‐MSC were attributed to the improved LAP and anti‐bacterial activities of macrophages, without affecting immunologic defense against pathogens and homeostasis of microbiota. Therefore, we conclude that BM‐MSC is a superior therapy for prevention of post‐stroke pneumonia.

Therapeutic efficacy of BM‐MSC in pneumonia has been demonstrated by accumulative studies.^[^ ^31^, ^32^ ^]^ However, the underlying cellular and molecular mechanisms remain to be elusive. As for the intrinsic bactericidal effects of BM‐MSC, we found that BM‐MSC rarely engulfed bacteria, which coincided with previous report.^[^ ^33^, ^34^ ^]^ We recorded that BM‐MSC released migrasomes that contained multiple anti‐bacterial peptides, including DCD, when encountering bacterial stimulus. Nevertheless, DCD‐containing migrasomes failed to suppress bacterial growth or exert bacteriolytic functions.

According to our data, the anti‐bacterial effects of BM‐MSC treatment could be attributed to their immunomodulatory functions. As indicated by RNA‐seq of the lung from tMCAO mice, BM‐MSC transfer facilitated the bactericidal activities of macrophages by improving their phagocytosis and digestion of bacteria. It has been reported that BM‐MSC shift macrophage toward M2 phenotype,^[^ ^35^ ^]^ which is endowed with prominent phagocytic capability.^[^ ^36^ ^]^ LC3‐associated phagocytosis (LAP), a non‐canonical autophagic pathway, is responsible for bacteria killing in macrophages.^[^ ^17^, ^37^ ^]^ The current study demonstrated that adoptive BM‐MSC transfer enhance LAP of macrophages, which is indispensable for the anti‐bacterial effect of the treatment.

Although BM‐MSC efficiently down‐regulated pulmonary bacterial load of tMCAO mice. The residence time of BM‐MSC in the lung was short. The anti‐bacterial effects of adoptive BM‐MSC were prominent at 3d after stroke and seemed to be retained until 14d. Nevertheless, in vivo tracking of BM‐MSC revealed that they infiltrated the lung shortly after tMCAO and soon retreated within 24 h. It was the extracellular vesicles, specifically, migrasomes, that were left in the lung and exerted the immunol‐modulating and anti‐bacterial functions. Migrasomes are migrating cells derived large vesicles that contain small vesicles which are Tetraspanin (TSPAN) 4‐expressing membrane‐bound structures that bind to retraction fibers.^[^ ^38^ ^]^ Retention of migrasomes in the lung of BM‐MSC transferred stroke models was documented for at least 3d after tMCAO, which, as far as we were concerned, were produced by migrating BM‐MSC when passing the lung.

DCD, the anti‐bacterial peptide that was first discovered in human sweat,^[^ ^24^ ^]^ was packed in BM‐MSC‐derived migrasomes. To be noticed, although DCD expression and its loading in migrasomes increased after bacterial stimulation, the anti‐bacterial peptide was constitutively produced by BM‐MSC. We found that DCD was indispensable for the LAP‐enhancing effects of BM‐MSC to pulmonary macrophages. However, DCD that was packed in migrasomes displayed more prominent LAP improvement than that was directly treated to macrophages. Considering the critical roles of macrophages and their LAP in anti‐pneumonia immune responses, we reason that DCD‐containing migrasome is a promising therapy for post‐stroke pneumonia.

## 4 Conclusion

BM‐MSC transfer protects against post‐stroke pneumonia through releasing DCD‐containing migrasomes, which enhance LAP of pulmonary macrophages. BM‐MSC not only displays efficient neuronal protection but also exert anti‐bacterial and immunol modulatory functions, and is thus a potential therapeutic treatment for AIS and post‐stroke pneumonia, which is more than a match for antibiotics.

## 5 Experimental Section

### Ethics Approval

All animal experiments were approved by the Third Affiliated Hospital of Sun Yat‐sen University and performed following the Guide for the Care and Use of Laboratory Animals and Stroke Treatment. Clinic research was approved by the ethics committee of the Third Affiliated Hospital of Sun Yat‐sen University.

### Human Material

Peripheral blood samples were provided by 8 healthy donors and 16 stroke patients recruited in the Third Affiliated Hospital of Sun Yat‐sen University from July 2018 to October 2020 consecutively at 0–3d after stroke onset. Demographic characteristics of the AIS patients and healthy controls were summarized in Table S1 (Supporting Information). Magnetic resonance imaging (MRI) was performed within 24 h of admission using 1.5‐ or 3.0‐T magnetic resonance imaging (Sigma; GE Medical Systems, Milwaukee, WI, USA). In this study, the diffusion‐weighted imaging (DWI) lesions in 16 patients were measured with Analyze 7.0 software (Analyze Direct, KS). Cerebral infarct size was assessed based on the largest infarct diameter determined on the image demonstrating the largest lesion. Plasma of AIS patients was collected via centrifugation of peripheral blood (15 000 g, 15 min, 4°C) and stored at −80°C before examination.

### Isolation and Identification of Human Bone Marrow Mesenchymal Stem Cells (BM‐MSC)

Heparin‐treated bone marrow was obtained from healthy donors along with their informed consent. Protocols for the derivation of BM‐MSC have been described previously.^[^ ^39^ ^]^ Briefly, BM‐MSC were separated by Ficoll–Paque (1.077 g ml^−1^, 17‐1440‐02, GE) density gradient centrifugation from the bone marrow and cultured with MSC SFM basal medium (GBICO, A13829‐01) plus MSC SFM supplement (GBICO, A11577‐01). The BM‐MSC were identified by exhibiting the surface characteristics of CD29 and CD90 (MSC markers) but not CD34 and CD45 (hematopoietic markers).

### Osteogenic and Adipogenic Differentiation of Bone Marrow Mesenchymal Stem Cells

The multi‐differentiation potential of BM‐MSC was investigated. The osteogenic medium consisting of ascorbic acid (50 µm, sigma, 2 043 003), dexamethasone (10 nm, Sigma, D4902), and *β*‐glycerophosphate (10 mm, Sigma, G9422) was used for BM‐MSC osteogenic differentiation and refreshed every 3 days for 21 days. The adipogenic medium contained 3‐isobutyl‐1‐methylxanthine (0.5 mm, Sigma, I5879), insulin (5 µg ml^−1^, Sigma, I2643), indomethacin (200 µm, Sigma, I7378) and dexamethasone (1 µm, Sigma, D4902) was used for BM‐MSC adipogenic differentiation and refreshed every 3 days for 14 days. The ability of BM‐MSC to differentiate into osteogenic and adipogenic cells was assessed by Alizarin Red S (Solarbio, G8550) and Oil Red O (Solarbio, G1260) staining, respectively.^[^ ^40^ ^]^

### Animals

C57/Bl6 wild‐type mice were purchased from Guangdong Medical Laboratory Animal Center (Guangzhou, China). All animals were housed in the animal facility in Sun Yat‐sen University. In the housing facility, humidity and temperature were controlled, and a 12 h light‐dark cycle was set. Water and food were freely available. Animals were housed in the animal facility for at least 1 week before induction of ischemic stroke. Male mice that were 8–10 weeks old with a weight of 18–25 g were included in the in vivo experiments.

### Murine Models of Acute Cerebral Ischemic Stroke

An ischemic stroke model was induced with transient middle cerebral artery occlusion (tMCAO) as previously described.^[^ ^36^ ^]^ In brief, mice were anesthetized with 1.5–2.0% isoflurane under spontaneous breath. A filament was inserted into the external carotid artery (ECA) and was directed to the middle cerebral artery (MCA) through the internal carotid artery (ICA). Silicon‐coated nylon filaments with various diameter were determined by the body weight of each mouse (weight 19–20.5 g, silicon diameter of 0.21±0.02 mm; weight 20.5–22 g, silicon diameter of 0.22±0.02 mm; weight 22.5–24 g, silicon diameter in 0.23±0.02 mm; weight 24–25 g, silicon diameter in 0.24±0.02 mm). Filaments were left in the MCA for 60 min. Cerebral reperfusion was fulfilled after the filament was retracted. During the surgery, body temperature of mice was monitored and maintained with a heating pad. Regional cortical cerebral blood flow (rCBF) was monitored before ischemia, during ischemia, and 15 min after reperfusion. rCBF was similar among the PBS‐, antibiotics‐ and MSC‐treated mice throughout the surgery and during reperfusion, which suggested that the extent of ischemic insult and blood flow recovery were similar among the mice with different treatments (Figure S1D, Supporting Information). Mice with more than 70% reduction of blood flow in the ischemic core were included in the study and mice that died during surgery were excluded. Sham‐operated animals underwent a similar procedure except for filament insertion. All efforts were made to minimize animal suffering.

### BM‐MSC Transplantation and Drug Administration in Mice

For BM‐MSC transplantation, 2 × 10^6^ cells (passages 5–8) were suspended in 0.1 ml PBS and transplanted via the inner canthal orbital vein at 2 h after MCAO. Broad‐spectrum antibiotic enrofloxacin was dissolved in sterile PBS and intraperitoneally (i.p.) administered (20 mg per kg per day) to mice for 3 consecutive days starting at 2 h post‐reperfusion. Mice in the Sham‐operated groups received equal volumes of PBS (i.p.) and served as control groups.

### Neurological Function Assessment

At 0–14d after tMCAO, the neurologic functions were assessed using the neurological deficit score and the modified Garcia score system as described previously.^[^ ^41^ ^]^ Neurological deficit scores were determined on a 0–4 scale: 0 = no apparent deficit, 1 = weakness in the ipsilateral forelimb (right), 2 = circulating to the ipsilateral side (right), 3 = body unbalance and trunk incline to ipsilateral, and 4 = no spontaneous motor activity or death.

### Behavior Tests

Sensorimotor functions of the stroke models were measured by rotarod test, adhesive‐removal test, and foot‐fault test at 1–3d before and 3–14d after tMCAO. In the rotarod test, mice were pre‐trained for three consecutive days before tMCAO using an accelerated paradigm. Data obtained at the last training test were recorded as a baseline. Tests were performed at 3d, 5d, 7d, 10d and 14d after cerebral ischemia. Mice were placed on a rotating drum with a speed accelerating from 0 to 50 rpm within 5 min and maintained at the final speed. The trial was ended if mouse fell off the drum or gripped the device and spun around for three consecutive revolutions. The latency to fall or spin around on the rung was recorded. Three trials were performed per testing day and the mean values were expressed as data. The adhesive removal test was performed to assess tactile responses and sensorimotor asymmetries. A 2×3 mm adhesive tape was applied to lesioned forepaws. Tactile responses were measured by recording the time to remove the adhesive tape. The maximum observation period was 120 s. In each training/testing day, three trials were performed with each mouse. The mean values were recorded as data. Mice were pre‐trained for 3 consecutive days before tMCAO and data in the last straining session were set as baseline. Tests were performed at 3d, 5d, 7d, 10d and 14d after tMCAO. In foot‐fault test, a stainless‐steel grid floor (20 cm × 40 cm with a mesh size of 4cm^2^) was used as the testing field. Each mouse was placed on the grid floor and steps of mice were videotaped for 1 min. The number of total steps and forelimb foot‐fault (when the forelimb fell through the grid) was recorded. Mice were pre‐trained for three consecutive days before tMCAO and data in the last training session were set as baseline. Tests were performed at 3d, 5d, 7d, 10d, and 14d after tMCAO.

### Infarct Volume Analysis

For immunologic staining of NeuN, six equally spaced coronal brain sections encompassing the MCA territory were stained with NeuN antibodies. Infarct volume in NeuN‐stained sections was analyzed with NIH ImageJ software. The infarct area was determined as the difference between the NeuN‐positive area of contralateral hemispheres and ipsilateral hemispheres. Brain infarct was determined by multiplying the mean area of tissue loss by the distances between two adjacent stained brain slices. For staining of 2,3,5‐triphenyltetrazolium chloride (TTC, Sigma, T8877), brain was isolated and cut into sections in mold (1 mm thick). The brain slices were then stained with 2% TTC dissolved in PBS. The area of infarct lesion was determined as the difference between the TTC stained area (red) of contralateral hemisphere and ipsilateral hemisphere in each section. Brain infarct volume was determined by multiplying the mean area of tissue loss by the distances between the two adjacent stained brain slices.

### Collection of Bronchoalveolar Lavage Fluid (BALF)

In in vivo experiments, mice were sacrificed after tMCAO. The trachea was exposed and a cannula was inserted just below the larynx. The proximal end of the trachea was held around the cannula with forceps while 1.0 ml of sterile PBS was instilled into the lungs and recovered by aspiration. A total of 2.0 ml was introduced to the lungs. The BALF was centrifuged at 500 g to collect cells. The supernatant of BALF recovered was frozen at −80°C to later test for cytokines.^[^ ^42^ ^]^

### Primary Mouse Bone Marrow‐Derived Macrophage (BMDMs) Culture

Bone marrow cells were isolated from femurs and tibia of healthy WT C57/BL6 wild‐type donors (8–12 weeks old). Macrophage precursors were cultivated and differentiated into macrophages for 6 days with MCSF (50 ng ml^−1^) for 6 days in macrophage culture medium (RPMI1640 + 10%FBS).

### Human Monocyte Enrichment and Macrophage Differentiation

Mono‐nucleus cells were isolated from peripheral blood of healthy adults (age = 18–40y) with human peripheral blood monocyte isolation Solution kit (Solarbio, P8680). For macrophage differentiation, monocytes adhered 4 h on tissue‐culture plates were cultured in RPMI medium 1640 containing 10% FBS, 1% L‐glutamine, and 1% penicillin/streptomycin, 10% human serum and 30 ng ml^−1^ macrophage colony‐stimulating factor (M‐CSF, Peprotech, 300–25). Medium was replaced every 3 days until day 7 when the cells were prepared for further experiments.^[^ ^43^ ^]^

### Macrophage Depletion

Hematogenous macrophages and circulating monocytes were depleted with Clodronate liposomes (Clodrosome Macrophage Depletion Kit. LIPOSOMA, CP‐010‐010). Wild‐type C57/Bl6 mice were injected with Clodronate liposomes or control/empty liposomes at 72 h before tMCAO (75 mg kg^−1^, i.v). The efficiency of macrophage depletion was assessed with flow cytometry.

### Bulk RNA Sequencing and Data Analysis

The RNA of lung tissue was used to perform bulk RNA sequencing (RNA‐seq). RNA‐seq libraries were generated using the NEBNext® Ultra™ RNA Library Prep Kit for Illumina. After qualification, the different libraries were sequenced on Illumina NovaSeq 6000 platform. For data analysis, *p* < 0.05 and |log2(foldchange)| >0 were set as the threshold for significantly differential expression. The “DESeq2” and the “ClusterProfiler” packages of the R software were used for differential analysis and enrichment analysis (Gene ontology and Reactome pathway analysis), respectively.

### Histological Analyses

Lung was fixed in 4% paraformaldehyde and embedded in paraffin. Lung sections were cut into 8 µm in thickness sections and were deparaffinized, rehydrated, and stained with hematoxylin and eosin (H&E) for histological analyses.

### Fluorescent in Situ Hybridization (FISH)

Lung tissue sections of stroke mice were processed for fluorescent in situ hybridization (FISH) was performed with Cy3 labeled EUB338 FISH probe (focobio, EUB338) and fluorescent in situ hybridization kit (focobio, D‐0016), following the manufacturers' protocols.

### Isolation and Characterization of Extracellular Vesicles (EVs) and Migrasomes from BM‐MSC

The isolation of EVs was performed from culture supernatants of BM‐MSCs. After BM‐MSC cultured to reach 70–80%, BM‐MSC culture supernatants were collected. To remove cells and large debris, BM‐MSC culture supernatants were centrifuged at 1000 g for 10 min and 4000 g for 20 min. The supernatant was finally centrifuged at 100 000 g for 70 min to obtain the BM‐MSC‐derived EVs.^[^ ^23^ ^]^ To collect BM‐MSC‐derived migrasomes, BM‐MSCs were first cultured in dishes coated with fibronectin (0.1 µg ml^−1^, BD, 354 008). When the cell density reached 50%, digestion was performed with 0.125% trypsin. Cells and large debris were removed by the centrifugation at 1000 g for 10 min and subsequently 4000 g for 20 min, respectively. The supernatant was finally centrifuged at 20 000 g for 30 min to obtain the migrasomes.^[^ ^20^, ^44^ ^]^ To exclude migrasomes in the EVs, the digested culture were subjected to serial centrifugation of 1000 g, 10 min; 4000 g, 20 min and 20 000 g, 30 min. The supernatant was finally centrifuged at 100 000 g for 70 min to obtain BM‐MSC‐derived EVs which migrasome were removed (EVs‐migrasomes) (Figure S7D, Supporting Information). Before further analysis or in vitro treatment, crude pellets were washed with PBS and centrifuged two times at 20 000 g for 30 min (migrasomes) or 100 000 g for 70 min (EVs and EVs‐migrasomes). Transmission electron microscopy (TEM) was used to observe the morphology of extracted migrasomes. The particle size and zeta potential of EVs‐migrasomes and migrasomes were detected by the Particle Analyzer Litesizer 500 (Anton Paar, Austria). The concentrations of vesicles were determined by protein content and western blot was conducted to detect surface markers of EVs and migrasomes.

### Liquid Chromatography Tandem Mass Spectrometry (LC‐MS/MS) Analysis

Liquid chromatography tandem mass spectrometry (LC‐MS/MS) was performed to profile the proteome of migrasomes. Migrasome proteins were separated by SDS‐PAGE and stained with Coomassie brilliant blue. The obtained gels were then processed through the in‐gel trypsin digestion. Next, tandem mass spectrometry data were acquired on a Q Exactive mass spectrometer (ThermoFisher) equipped with a Nano Flex ion source. For protein identification and quantification, analysis was performed with PEAKS Studio software (version 8.5).

### Negative Staining Transmission Electron Microscopy (TEM) Analysis

Drops of EVs (4–8 µl) in PBS were adsorbed at activated Formvar/Carbon coated grids for 3–15 min at room temperature (RT) and subsequently stained at RT. Samples were stained either with 2% ammonium molybdate for 20–30s. EVs were examined with a TEM of HITACHI HT7800/HT7700.

### Phagocytosis Assay

For evaluation of efferocytic capacity, *E. Coli* labeled with GFP or red *E. Coli* (Biovision, K964‐100) were treated with macrophages at a ratio of dead *E. Coli* : BMDM = 20:1, for the indicated time periods. For in vitro immunostaining experiments, BMDM were pre‐grown on poly‐l‐lysine‐coated coverslips. The coverslips of BMDM were washed two times to remove unengulfed *E. Coli* and fixed with 4% paraformaldehyde. The coverslips were then subjected to immunostaining and removed from wells using tweezers and mounted to the slides. F‐actin in *E. Coli* was then stained with Alexa Fluor 488 phalloidin (Invitrogen, A12380, 1:3000) at room temperature in the dark for 30 min. For the flow cytometry experiment, BMDM were presided on 24‐well plates and treated with the same ratio of dead neurons for indicated time points. BMDM were washed with PBS, detached from wells with trypsin, and subjected to flow cytometric analysis.

### In Vivo MSC Tracing Experiments

For in vivo tracing, BM‐MSC was labeled with DIR (Meilunbio, MB12482‐1) before infusion. Mice were sacrificed at 2 h, 1d and 3d after intravenous infusion, respectively. The fluorescence intensity of major organs was then detected by AniView600 (BLT).

### Lentiviral Infection of BMDM and BM‐MSC

To knockdown the expression of RUBCN in BMDM and DCD in BM‐MSC, shRNA of RUBCN or DCD was inserted into the lentiviral transfer vector, respectively. Four shRNA were designed and two efficiently knocked down the expression of RUBCN or DCD, whose sequences were: Rubcn KD2: 5′‐CCGGCTGGCAGCTGCTGGGTAATTTCTCGAGAAATTACCCAGCAGCTGCCAGTTTTTT‐3′, Rubcn KD4: 5′‐CCGGGTAAAGCGTGTTACCATAAAGCTCGAGCTTTATGGTAACACGCTTTACTTTTTT‐3′; DCD KD1: 5′‐GATCTAGAAAGCGTGGGTAAACTCGAGTTTACCCACGCTTTCTAGATC–3′, DCD KD3:5′‐TTAGCCAGACAGGCACCAAAGCTCGAGCTTTGGTGCCTGTCTGGCTAA‐3′. The constructed transfer vectors were transformed into DH5*α E. Coli* and then isolated using the Endo‐free Plasmid Mini Kit (Omega, D6950). A plasmid mixture containing pSPAX2, pMD2.G, and vector was suspended in OPTI‐MEM (GBICO, 31 985 070), and PEI MAX 25K (Polysciences, 23 966) was applied as transfection reagent. The plasmids containing OPTI‐MEM were added to 293T cells and incubate for 4 h before switching to fresh medium. At 48 h after transfection, the supernatant was centrifuged at 800 g for 10 min to remove debris from the cells. The lentivirus‐containing medium was treated to MSC for 48 h to fulfill shRNA dependent knock down. The efficiency was finally evaluated by RT‐PCR or western blot.

### Immunofluorescence Staining

In in vivo experiments, mice were sacrificed at 3d or 14d after tMCAO. After sufficient perfusion with 10 ml of PBS and 10 ml of 4% paraformaldehyde, brains were cut into coronal sections (25 µm), lung sections were cut into sections (8–12 µm) on a frozen microtome. In in vitro experiments, BMDM or BM‐MSC were seeded on coverslips coated with poly‐L‐lysine (Sigma, P2636). After treatment, cells were fixed with 4% paraformaldehyde. Brain sections, lung sections, or fixed BMDM, BM‐MSC were washed and incubated with primary antibodies overnight in PBS containing 0.03% Triton‐X100 and 3% BSA. After washing, sections or cells were incubated with secondary antibodies for 1 h at room temperature. The following primary antibodies were used: rabbit anti‐NeuN (Abcam, ab177487, 1:300), mouse anti‐MBP (Merck millipore, MABT1499, 1:300), mouse anti‐NF‐H (Proteintech, 60331‐1‐Ig, 1:300), goat anti‐Iba1 (Abcam, ab5076, 1:300), rabbit anti‐RUBCN (Proteintech, 21444‐1‐AP, 1:300), rabbit anti‐NAG‐2 (Abcam, ab181995, 1:300), rabbit anti‐DCD (Novus, NBP2‐92673, 1:300), mouse anti‐BECN1 (Proteintech, 11303‐1‐Ap, 1:300), rabbit anti‐TREM2 (Abcam, ab86491, 1:300) and rabbit anti‐TIMD4 (Proteintech, 12008‐1‐AP,1:300). The following secondary antibodies were applied: anti‐rabbit secondary antibody conjugated with Cy3 (Jackson ImmunoResearch Laboratories, 115‐165‐003, 1:1000), anti‐mouse secondary antibody conjugated with Alexa Fluor 488 (Jackson ImmunoResearch Laboratories, 112‐545‐003, 1:1000), anti‐goat secondary antibody conjugated with Alexa Fluor 488 (Jackson ImmunoResearch Laboratories, 705‐545‐003, 1:1000), and anti‐rabbit secondary antibody conjugated with Alexa Fluor 647 (Jackson ImmunoResearch Laboratories, 111‐605‐003, 1:1000. Phalloidin (Invitrogen, A12380, 1:3000) was used to label F‐actin. Wheat germ agglutinin (WGA, Invitrogen W7024, 1 µg ml^−1^) which was a widely used lectin that binds to sialic acid and N‐acetylglucosaminyl residues in cell membrane was used to label cell membrane. DAPI Fluoromount‐G (Southern Biotech) was applied to locate nucleus when indicated. For the TUNEL staining, Click‐iT Plus TUNEL 488 (Invitrogen, C10617) was used according to manufacturers instructions.

### Real‐Time Polymerase Chain Reaction (RT‐PCR)

Total RNA from cells was extracted with commercial kit (ESscience, RN001) according to the manufacturer' s instructions. A total of 1 µg RNA (OD260nm/280 nm = 1.8–2.2) was used for the first strand cDNA synthesis in a 20ul system using Fast Reverse Transcription kit (ESscience, RT001). Real time polymerase chain reaction (RT‐PCR) was carried out using 1 µl of the synthesized cDNA per reaction with the addition of SYBR Green qPCR Mix (Dongshengbio, P2092a) on the QuantStudio 5 (ABI) quantitative PCR machine. The program was as follows: 95°C for 30s; 95°C for 5 s and 60°C for 34s, repeated for 40 cycles; 95°C for 15 s, 60°C for 1 min and 95°C for 15 s (Melt curve). Human and mouse primer sequences were listed in Table S2 (Supporting Information). For 16S rRNA analysis, the universal bacteria primers 27F (5′‐AGAGTTTGATCCTGGCTCAG‐3') and 1492R (5″‐GCTTACCTTGTTACGACTT‐3″) were used. Delta CT log2 (compared with the CT value of Gapdh) was calculated and normalized to the means of control group.

### Immunoprecipitation (IP)

Protein of 5 × 10^6^ BMDM was extracted with 1 ml of RIPA lysis buffer (Sigma, R0278). Antibodies (2 µg per sample) were applied to the IP system and incubated overnight at 4°C on a shaker. Pierce protein A/G magnetic beads (Thermo Fisher, 88 802) were then added into the system (10 µl per sample). The IP system was then incubated in a rotator for additional 1–2 h at 4°C. After three washes with 1 ml RIPA lysis buffer, 100 µl of HCLGlycine (pH = 2.0) was applied to elute the precipitated protein. The sample was then subjected to western blot experiments. The following primary antibodies were used: rabbit anti‐RUBCN (Proteintech, 21444‐1‐Ap), and mouse anti‐BECN1 (Proteintech 11303‐1‐Ap).

### Western Blot

Protein was extracted with RIPA lysis buffer (Beyotime, P0013). A total amount of 40 µg protein of each sample was applied to western blot experiments. Western blot was performed with standard SDS‐polyacryamide gel electrophoresis (SDS‐PAGE) method and enhanced chemiluminescence detection reagents (Invitrogen). The following primary antibodies were used: rabbit anti‐SQSTM1 (Proteintech, 18420‐1‐Ap, 1:2000), rabbit anti‐LC3A/B (Cell signaling technology, 12741T, 1:5000), rabbit anti‐RUBCN (Proteintech, 21444‐1‐Ap, 1:300), mouse anti‐BECN1 (Proteintech 11303‐1‐Ap, 1:300), rabbit anti‐ATG3 (Cell signaling technology, 3415T, 1:1000), rabbit anti‐ATG5 (Cell signaling technology, 12994T, 1:1000), rabbit anti‐ATG7 (Cell signaling technology, 8558T, 1:1000), rabbit anti‐ATG12 (Cell signaling technology, 4180T, 1:1000), rabbit anti‐ATG14 (Proteintech, 19491‐1‐AP, 1:1000), rabbit anti‐ATG16L (Cell signaling technology, 8089T, 1:1000), rabbit anti‐C3ORF64 (Proteintech, 27595‐1‐AP, 1:1000), rabbit anti‐PIGK (Proteintech, 27595‐1‐AP, 1:1000), rabbit anti‐PGCP (Proteintech, 16601‐1‐AP, 1:1000), rabbit anti‐NDST1 (Proteintech, 26203‐1‐AP, 1:1000), rabbit anti‐NAG‐2 (Abcam, ab181995, 1:1000), rabbit anti‐TSPAN7 (Proteintech, 18695‐1‐AP, 1:1000), rabbit anti‐TSPAN9 (Proteintech, 21983‐1‐AP, 1:1000), rabbit anti‐HSP70 (Affinity, AF5466, 1:1000), mouse anti‐HSP90 (Proteintech, 60318‐1‐Ig, 1:5000), and mouse anti‐GAPDH (Proteintech, 60004‐1‐Ig, 1:10 000).

### Flow Cytometric Analysis

In in vivo experiments, mice were sacrificed after tMCAO. The BALF was centrifuged at 500 g to collect cells. After being washed with PBS, cells were fixed and permeabilized (Invitrogen, Intracellular Fixation & Permeabilization Buffer Set), then stained with intracellular antibodies. The following antibodies were used: anti‐CD45‐BV421 (Biolegend, 103 134, clone: 30‐F11, 1:400), anti‐ F4/80‐AF488 (Biolegend, 123 120, clone: BM8, 1:400), anti‐LY6G‐APCCy7 (Biolegend, 108 424, clone: RB6‐8C5, 1:400), anti‐CD11c‐APC (Biolegend, 117 310, clone: N418, 1:400), anti‐CD3‐PECy7 (Biolegend, 100 220, clone: 17A2, 1:400), anti‐CD19‐PE (Biolegend, 152 408, clone: 1D3/CD19, 1:400), anti‐CD45‐PerCPCy5.5 (Biolegend, 368 504, clone: 2D1, 1:400), anti‐CD90‐FITC (Biolegend, 328 108, clone: 5E10, 1:400), anti‐CD34‐APC (Biolegend, 343 509, clone: 581, 1:400), and anti‐CD29‐PE (Biolegend, 303 004, clone: TS2/16, 1:400). Cell viability of BMDM was assessed using the Annexin V‐APC/PI apoptosis Detection Kit (KeyGEN, KGA1030‐100) according to manufacturer' s instructions.

### Enzyme‐Linked Immunosorbent Assay (ELISA)

The concentrations of DCD in BM‐MSC, migrasomes, or plasma of AIS patients were measured using the human DCD ELISA kit (FineTest, EH4251). The LPS level of BALF of tMCAO mice was quantified using the mouse LPS ELISA kit (MEIMIAN, MM‐0634M1). ELISA was carried out using plasma samples according to the manufacturer's instructions.

### Statistics

GraphPad Prism software (version 9.0) was used for statistical analysis. Results were presented as mean ± standard deviation (SD) or mean ± standard error of mean (SEM). Student's *t* test was performed in data comparison of two groups. One‐way ANOVA was performed in data comparison of three groups or more. Log‐rank test was performed on survival rates. To assess the associations of indications, Spearman correlation analysis was applied to assess the correlations of continuous variables, and Point‐biserial correlations analysis was applied to assess correlations between dichotomous and continuous variables. Results were considered significant at *p* < 0.05.

## Conflict of Interest

The authors declare no conflict of interest.

## Author Contributions

T.L., X.S., and P.L. contribute equally to the study. T.L., X.S., and P.L. performed the experiments. X.K. and M.H. collected and analyzed data and contributed to the experimental design. C.L. and S.W. participated in animal experiments. D.L., S.S., H.H., and Y.L. collected samples from donors and participated in data analysis. X.D. participated in data analysis. W.C., L.W., and Z.L. designed and supervised the study and critically revised the manuscript. All authors have read and approved the final manuscript.

## Supporting information

Supporting Information

## Data Availability

The data that support the findings of this study are available in the supplementary material of this article.

## References

[1] W. F. Westendorp , C. Dames , P. J. Nederkoorn , A. Meisel , Stroke 2022, 53, 1438. 35341322 10.1161/STROKEAHA.122.038867

[2] J. Faura , A. Bustamante , F. Miró‐Mur , J. Montaner , J. Neuroinflamm. 2021, 18, 127. 10.1186/s12974-021-02177-0 PMC8183083 34092245

[3] S. Suda , J. Aoki , T. Shimoyama , K. Suzuki , Y. Sakamoto , T. Katano , S. Okubo , C. Nito , Y. Nishiyama , M. Mishina , K. Kimura , J. Neurol. 2018, 265, 370. 29249057 10.1007/s00415-017-8714-6

[4] A. Bustamante , D. Giralt , T. García‐Berrocoso , M. Rubiera , J. Álvarez‐Sabín , C. Molina , J. Serena , J. Montaner , Eur. Stroke J. 2017, 2, 54. 31008302 10.1177/2396987316681872 PMC6453178

[5] J.‐D. Vermeij , W. F. Westendorp , D. W. Dippel , D. van de Beek , P. J. Nederkoorn , Cochrane Database Syst. Rev. 2018, 1, CD008530. 29355906 10.1002/14651858.CD008530.pub3 PMC6491314

[6] N. A. Scott , A. Andrusaite , P. Andersen , M. Lawson , C. Alcon‐Giner , C. Leclaire , S. Caim , G. L.e Gall , T. Shaw , J. P. R. Connolly , A. J. Roe , H. Wessel , A. Bravo‐Blas , C. A. Thomson , V. Kästele , P. Wang , D. A. Peterson , A. Bancroft , X. Li , R. Grencis , A. M. Mowat , L. J. Hall , M. A. Travis , S. W. F. Milling , E. R. Mann , Sci. Transl. Med. 2018, 10, 464. 10.1126/scitranslmed.aao4755 PMC6548564 30355800

[7] J. H. Yang , P. Bhargava , D. McCloskey , N. Mao , B. O. Palsson , J. J. Collins , Cell Host Microbe 2017, 22, 6. 10.1016/j.chom.2017.10.020 PMC5730482 29199098

[8] A. Andrzejewska , S. Dabrowska , B. Lukomska , M. Janowski , Adv. Sci. 2021, 8, 2002944. 10.1002/advs.202002944 PMC8024997 33854883

[9] S. J. Turley , V. Cremasco , J. L. Astarita , Nat. Rev. Immunol. 2015, 15, 669. 26471778 10.1038/nri3902

[10] S. Dabrowska , A. Andrzejewska , B. Lukomska , M. Janowski , J. Neuroinflamm. 2019, 16, 178. 10.1186/s12974-019-1571-8 PMC6743114 31514749

[11] L. Wang , Z. Deng , Y. Zhao , R. Yuan , M. Yang , Y. Zhang , Y. Li , Y. Liu , F. Zhou , H. Kang , J. Therm. Biol. 2021, 101, 103081. 34879909 10.1016/j.jtherbio.2021.103081

[12] J. Park , S. Kim , H. Lim , A. Liu , S. Hu , J. Lee , H. Zhuo , Q. Hao , M. A. Matthay , J.‐W. Lee , Thorax 2019, 74, 43. 30076187 10.1136/thoraxjnl-2018-211576 PMC6295323

[13] L. Shi , H. Huang , X. Lu , X. Yan , X. Jiang , R. Xu , S. Wang , C. Zhang , X. Yuan , Z. Xu , L. Huang , J.‐L. Fu , Y. Li , Y. Zhang , W.‐Q. Yao , T. Liu , J. Song , L. Sun , F. Yang , X. Zhang , B. Zhang , M. Shi , F. Meng , Y. Song , Y. Yu , J. Wen , Q. Li , Q. Mao , M. Maeurer , A. Zumla , et al., Signal Transduct. Target Ther. 2021, 6, 58. 33568628 10.1038/s41392-021-00488-5 PMC7873662

[14] E. S. Kim , S. Y. Ahn , G. H. Im , D. K. Sung , Y. R. Park , S. H. Choi , S. J. Choi , Y. S. Chang , W. Oh , J. H. Lee , W. S. Park , Pediatr. Res. 2012, 72, 277. 22669296 10.1038/pr.2012.71

[15] M. S. V. Elkind , A. K. Boehme , C. J. Smith , A. Meisel , M. S. Buckwalter , Stroke 2020, 51, 3156. 32897811 10.1161/STROKEAHA.120.030429 PMC7530056

[16] D. L. Bonilla , A. Bhattacharya , Y. Sha , Y. Xu , Q. Xiang , A. Kan , C. Jagannath , M. Komatsu , N. T. Eissa , Immunity 2013, 39, 537. 24035364 10.1016/j.immuni.2013.08.026 PMC8059138

[17] S. Masud , T. K. Prajsnar , V. Torraca , G. E. M. Lamers , M. Benning , M. Van Der Vaart , A. H. Meijer , Autophagy 2019, 15, 796. 30676840 10.1080/15548627.2019.1569297 PMC6526873

[18] B. L. Heckmann , B. J. W. Teubner , B. Tummers , E. Boada‐Romero , L. Harris , M. Yang , C. S. Guy , S. S. Zakharenko , D. R. Green , Cell 2019, 178, 3. 10.1016/j.cell.2019.05.056 PMC6689199 31257024

[19] J. Wan , E. Weiss , S. Ben Mkaddem , M. Mabire , P.‐M. Choinier , O. Picq , T. Thibault‐Sogorb , P. Hegde , D. Pishvaie , M. Bens , L. Broer , H. Gilgenkrantz , R. Moreau , L. Saveanu , P. Codogno , R. C. Monteiro , S. Lotersztajn , Sci. Transl. Med. 2020, 18, 12. 10.1126/scitranslmed.aaw8523 32295902

[20] L. Ma , Y. Li , J. Peng , D. Wu , X. Zhao , Y. Cui , L. Chen , X. Yan , Y. Du , L. Yu , Cell Res. 2015, 25, 24. 25342562 10.1038/cr.2014.135 PMC4650581

[21] V. Go , B. G. E. Bowley , M. A. Pessina , Z. G. Zhang , M. Chopp , S. P. Finklestein , D. L. Rosene , M. Medalla , B. Buller , T. L. Moore , Geroscience 2020, 42, 1. 31691891 10.1007/s11357-019-00115-w PMC7031476

[22] T. R. Doeppner , J. Herz , A. Görgens , J. Schlechter , A.‐K. Ludwig , S. Radtke , K. de Miroschedji , P. A. Horn , B. Giebel , D. M. Hermann , Stem Cells Transl. Med. 2015, 4, 1131. 26339036 10.5966/sctm.2015-0078 PMC4572905

[23] S. Dabrowska , A. Andrzejewska , D. Strzemecki , M. Muraca , M. Janowski , B. Lukomska , J. Neuroinflamm. 2019, 16, 216. 10.1186/s12974-019-1602-5 PMC6852925 31722731

[24] B. Schittek , R. Hipfel , B. Sauer , J. Bauer , H. Kalbacher , S. Stevanovic , M. Schirle , K. Schroeder , N. Blin , F. Meier , G. Rassner , C. Garbe , Nat. Immunol. 2001, 2, 1133. 11694882 10.1038/ni732

[25] C. Meisel , K. Prass , J. Braun , I. Victorov , T. Wolf , D. Megow , E. Halle , H.‐D. Volk , U. Dirnagl , A. Meisel , Stroke 2004, 35, 2. 14684767 10.1161/01.STR.0000109041.89959.4C

[26] D. Amantea , F. Petrelli , R. Greco , C. Tassorelli , M. T. Corasaniti , P. Tonin , G. Bagetta , Front. Neurosci. 2019, 13, 1256. 31849581 10.3389/fnins.2019.01256 PMC6902046

[27] X.‐H. Dong , C. Peng , Y.‐Y. Zhang , Y.‐L. Tao , X. Tao , C. Zhang , A. F. Chen , H.‐H. Xie , EBioMedicine 2017, 24, 116. 28928014 10.1016/j.ebiom.2017.09.002 PMC5652002

[28] L. Ulm , S. Hoffmann , D. Nabavi , M. Hermans , B.‐M. Mackert , F. Hamilton , I. Schmehl , G.‐J. Jungehuelsing , J. Montaner , A. Bustamante , M. Katan , A. Hartmann , S. Ebmeyer , C. Dinter , J. C. Wiemer , S. Hertel , C. Meisel , S. D. Anker , A. Meisel , Front. Neurol. 2017, 8, 153. 28484421 10.3389/fneur.2017.00153 PMC5402305

[29] G. Ianiro , H. Tilg , A. Gasbarrini , Gut 2016, 65, 1906. 27531828 10.1136/gutjnl-2016-312297

[30] Y. Heianza , Y. Zheng , W. Ma , E. B. Rimm , C. M. Albert , F. B. Hu , K. M. Rexrode , J. E. Manson , L. Qi , Eur. Heart J. 2019, 40, 3838. 31216010 10.1093/eurheartj/ehz231 PMC6911167

[31] H. Qin , A. Zhao , Protein Cell 2020, 11, 707. 32519302 10.1007/s13238-020-00738-2 PMC7282699

[32] J. Chen , X. Zhang , J. Xie , M. Xue , L. Liu , Y. Yang , H. Qiu , Stem Cell Res. Ther. 2020, 11, 311. 32698911 10.1186/s13287-020-01826-0 PMC7374869

[33] H. Yagi , A. F. Chen , D. Hirsch , A. C. Rothenberg , J. Tan , P. G. Alexander , R. S. Tuan , Stem Cell Res. Ther. 2020, 11, 293. 32680544 10.1186/s13287-020-01807-3 PMC7367313

[34] A. É. Silva‐Carvalho , M. H. Cardoso , T. Alencar‐Silva , G. M. R. Bogéa , J. L. Carvalho , O. L. Franco , F. Saldanha‐Araujo , Pharmacol. Ther. 2022, 233, 108021. 34637839 10.1016/j.pharmthera.2021.108021

[35] D.‐I. Cho , M. R. Kim , H.‐y. Jeong , H. C. Jeong , M. H. Jeong , S. H. Yoon , Y. S. Kim , Y. Ahn , Exp. Mol. Med. 2014, 46, 70. 10.1038/emm.2013.135 PMC3909888 24406319

[36] W. Cai , M. Hu , C. Li , R. Wu , D. Lu , C. Xie , W. Zhang , T. Li , S. Shen , H. Huang , W. Qiu , Q. Liu , Y. Lu , Z. Lu , Autophagy 2023, 19, 4. 10.1080/15548627.2022.2116833 PMC10012925 36170234

[37] M. Inomata , S. Xu , P. Chandra , S. N. Meydani , G. Takemura , J. A. Philips , J. M. Leong , Proc. Natl. Acad. Sci. USA 2020, 117, 33561. 33376222 10.1073/pnas.2015368117 PMC7776987

[38] Y. Huang , B. Zucker , S. Zhang , S. Elias , Y. Zhu , H. Chen , T. Ding , Y. Li , Y. Sun , J. Lou , M. M. Kozlov , L. Yu , Nat. Cell Biol. 2019, 21, 10. 10.1038/s41556-019-0389-z 31435030

[39] X. Zhang , W. Huang , X. Chen , Y. Lian , J. Wang , C. Cai , L. Huang , T. Wang , J. Ren , A. P. Xiang , Mol. Ther. 2017, 25, 1434. 28454789 10.1016/j.ymthe.2017.04.004 PMC5475252

[40] H. Li , P. Liu , S. Xu , Y. Li , J. D. Dekker , B. Li , Y. Fan , Z. Zhang , Y. Hong , G. Yang , T. Tang , Y. Ren , H. O. Tucker , Z. Yao , X. Guo , J. Clin. Invest. 2017, 127, 1241. 28240601 10.1172/JCI89511 PMC5373872

[41] W. Cai , J. Wang , M. Hu , X. Chen , Z. Lu , J. A. Bellanti , S. G. Zheng , J. Neuroinflamm. 2019, 16, 175. 10.1186/s12974-019-1557-6 PMC6717357 31472680

[42] L. Dong , Y. Wang , T. Zheng , Y. Pu , Y. Ma , X. Qi , W. Zhang , F. Xue , Z. Shan , J. Liu , X. Wang , C. Mao , Stem Cell Res. Ther. 2021, 12, 4. 33407872 10.1186/s13287-020-02072-0 PMC7789736

[43] K.‐Y. Park , G. Li , M. O. Platt , Sci. Rep. 2015, 5, 13855. 26349896 10.1038/srep13855 PMC4563359

[44] Y. Chen , Y. Li , L. Ma , L. Yu , Methods Mol. Biol. 2018, 1749, 43. 29525989 10.1007/978-1-4939-7701-7_5

