# Supplementary material for: Bone Marrow Mesenchymal Stem Cell‐Derived Dermcidin‐Containing Migrasomes enhance LC3‐Associated Phagocytosis of Pulmonary Macrophages and Protect against Post‐Stroke Pneumonia
Source: Adv Sci (Weinh). 2023 May 28;10(22):2206432. doi: 10.1002/advs.202206432 (PMC10401184; doi:10.1002/advs.202206432)
Supplement: Supplementary file 1 — Supporting Information [file ADVS-10-2206432-s001.pdf]

ADVANCED SCIENCE
Open Access

Supporting Information

for *Adv. Sci.*, DOI 10.1002/advs.202206432

Bone Marrow Mesenchymal Stem Cell-Derived Dermcidin-Containing Migrasomes enhance LC3-Associated Phagocytosis of Pulmonary Macrophages and Protect against Post-Stroke Pneumonia

Tiemei Li, Xiaotao Su, Pinglan Lu, Xinmei Kang, Mengyan Hu, Chunyi Li, Shisi Wang, Danli Lu, Shishi Shen, Huipeng Huang, Yuxin Liu, Xiaohui Deng, Wei Cai, Lei Wei* and Zhengqi Lu**

Supporting Information

Bone Marrow Mesenchymal Stem Cell-derived Dermcidin-containing Migrasomes Enhance LC3-associated Phagocytosis of Pulmonary Macrophages and Protect against Post-stroke Pneumonia

Tiemei Li, Xiaotao Su, Pinglan Lu, Xinmei Kang, Mengyan Hu, Chunyi Li, Shisi Wang, Danli Lu, Shishi Shen, Huipeng Huang, Yuxin Liu, Xiaohui Deng, Wei Cai, Lei Wei*, Zhengqi Lu **

This file includes:

Supplementary Figures

Figures S1. Identification of BM-MSC and animal experiment design.

Figure S2. Clodronate liposomes deplete macrophages in mice.

Figure S3. Quantitative analysis of pulmonary immune cells after stroke.

Figure S4. LC3 associated phagocytosis (LAP) in macrophages plays a major antibacterial role.

Figure S5. BM-MSC transferred to stroke recipients release migrasomes in the lung.

Figure S6. BM-MSC promote bacterial clearance of macrophages through releasing migrasomes.

Figure S7. Effect of dermcidin (DCD) on phagocytosis and immune function of macrophages.

Supplementary Tables:

Table S1. Demographic characteristics of the AIS patients and healthy controls.

Table S2. Primers used in the study.

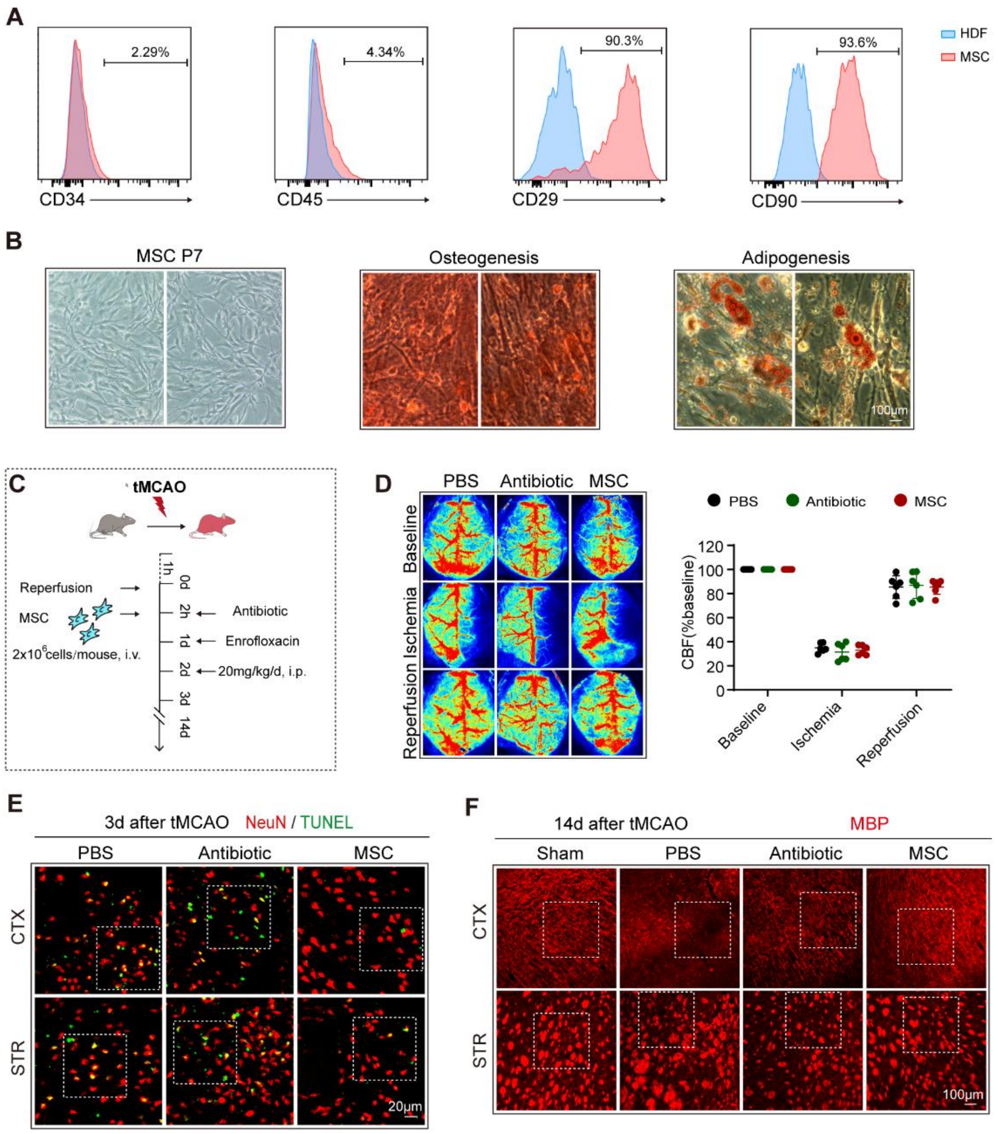

Figure S1. Identification of BM-MSC and animal experiment design.

(A) Examination of purity and stemness of BM-MSC. Experiments were repeated for three times. **(B)** Bright field images and examination of osteogenesis (Alizarin Red S) and adipogenesis (Oil Red O) of BM-MSC (P7) used in the study. Experiments were repeated for three times. **(C-D)** WT male C57/Bl6 mice were subjected to 60min of tMCAO, then treated with BM-MSC (2x10^6 cells per mouse, i.v.) at 2h after reperfusion or broad-spectrum antibiotic enrofloxacin (20mg/kg/ day, i.p) at 0-2d after reperfusion. Animals were sacrificed at 3- or 14-d after tMCAO. **(C)** Time line of the animal experiments was displayed. (**D**) No difference in regional cerebral blood flow (CBF) before ischemia, during ischemia and 15min after reperfusion among the 3 groups was detected. N = 6 in each group. (**E**) Neuronal injury in stroke penumbra was evaluated with co-immunostaining of NeuN (red) and TUNEL (green) at 3d after tMCAO. Representative images of $NeuN^+TUNEL^+$ neurons in stroke

penumbra of cortex (CTX) and striatum (STR) were displayed. *N* = 4-5 in each group. The enlarged images were shown in **Figure 1E**. (**F**) White matter integrity at 14d after tMCAO was analyzed with immunostaining of MBP. Representative images were displayed. *N* = 3-6 in each group. Enlarged Image have been shown in **Figure 1H**.

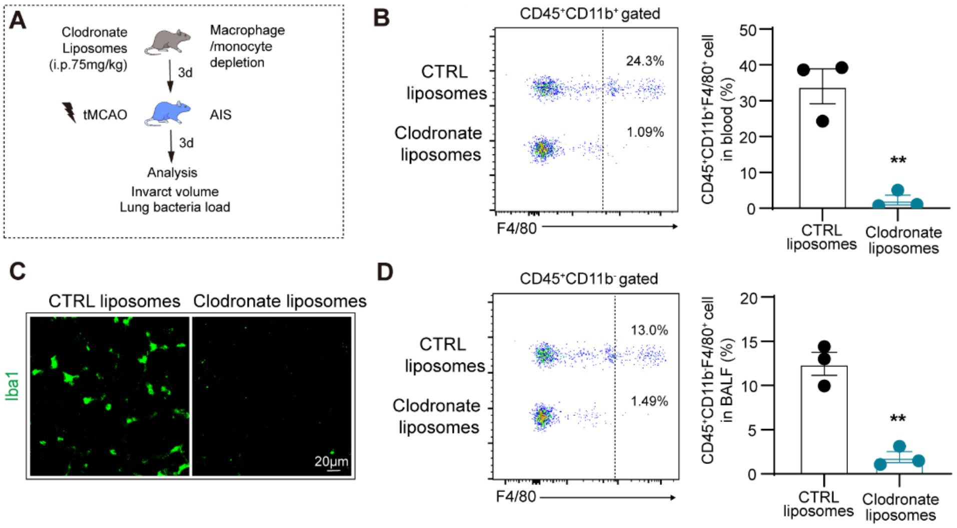

Figure S2. Clodronate liposomes deplete macrophages in mice.

(A) Wild type C57/Bl6 mice were injected with clodronate liposomes or control (CTRL) liposomes at 72h before tMCAO (75mg/kg, i.v.). **(B-D)** Impact of clodronate liposomes on *in vivo* macrophages in Sham-operated mice at 72h after injection. $N = 3$ mice per group. **(B)** Efficiency of blood macrophage depletion was assessed with flow cytometry. $^{**}P < 0.01$, compared with CTRL-liposomes group by Student's *t*-test (mean ± standard error). **(C-D)** Efficiency of clodronate liposomes on clearance of pulmonary macrophages was assessed with immunofluorescence staining **(C)** and flow cytometry **(D)**. $^{**}P < 0.01$, compared with CTRL-liposomes group by Student's *t*-test (mean ± standard error).

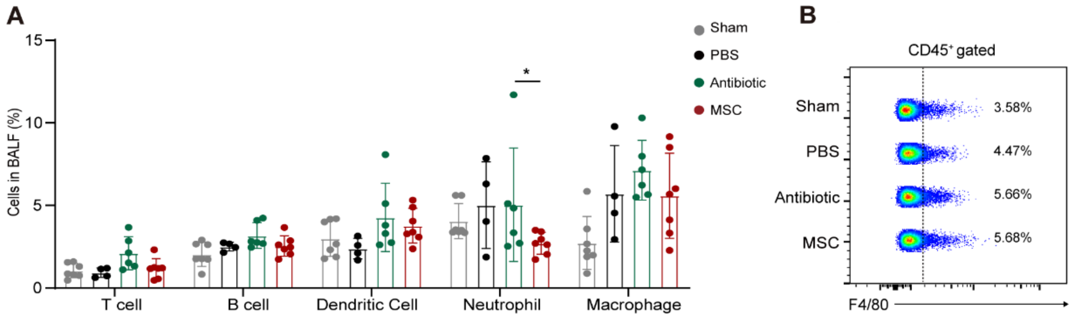

Figure S3. Quantitative analysis of pulmonary immune cells after stroke.

(A) Flow cytometry was used to quantify various immune cells in bronchoalveolar lavage fluid (BALF) of mice treated with PBS, antibiotics and BM-MSC at 3d after tMCAO. $N = 7$ in Sham-operated group, $N = 4$ in PBS-treated group, $N = 6$ in antibiotic-treated group, and $N = 7$ in BM-MSC-treated group. *$P < 0.05$, compared with antibiotic-treated group by one-way *ANOVA* (mean ± standard deviation). **(B)** Representative flow analysis plots of each group.

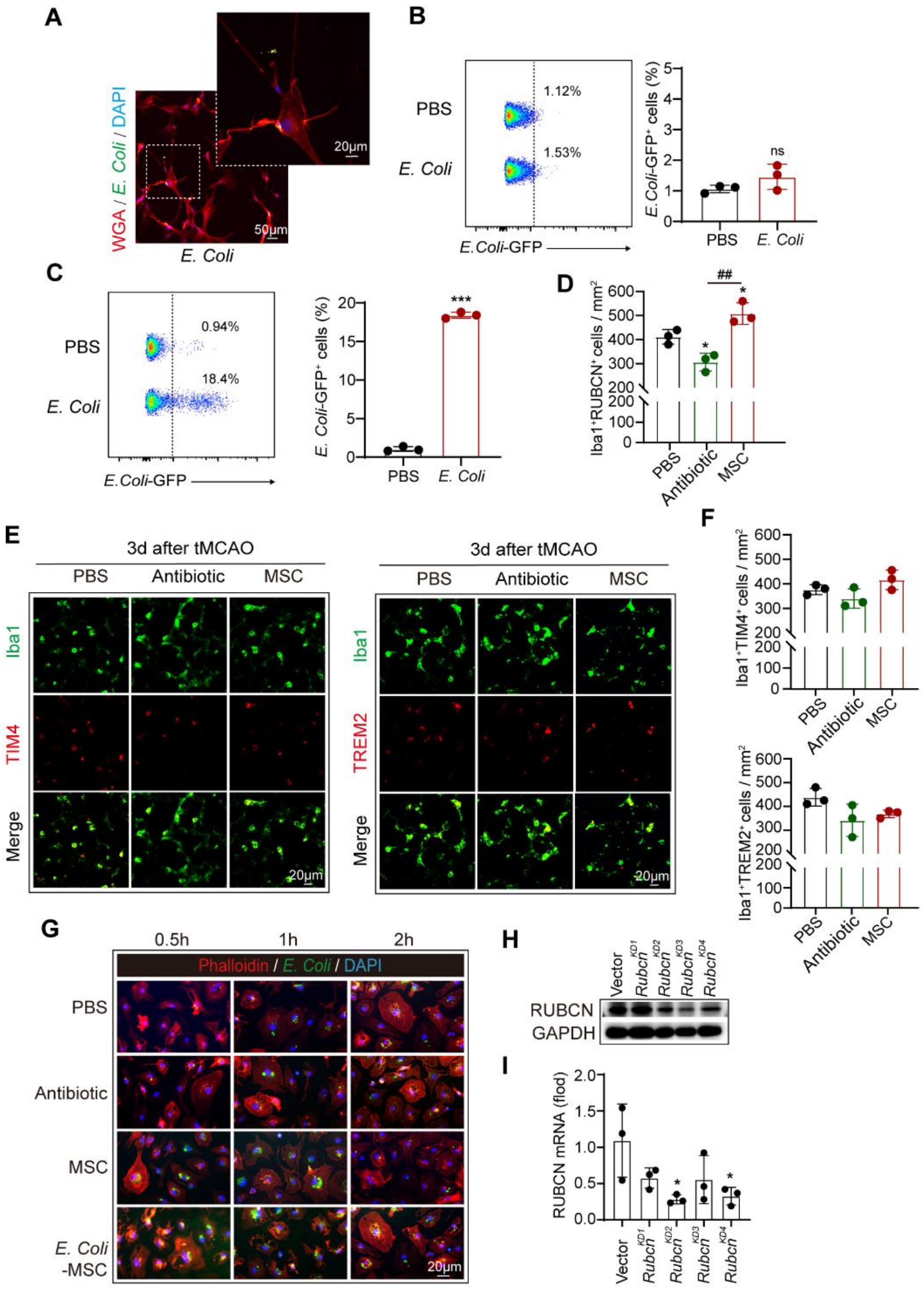

Figure S4. LC3 associated phagocytosis (LAP) in macrophages plays a major antibacterial role.

(A-B) BM-MSC were treated with *E. Coli* (*E. Coli* : BM-MSC = 20:1), and the phagocytosis of GFP expressing *E. Coli* was assessed at 1h with immunostaining **(A)** and flow cytometry **(B)**. Experiments were repeated for three times. **(C)** Phagocytosis of GFP expressing *E. Coli* by BMDM (*E. Coli* : BMDM = 20:1, 1h) was analyzed by flow cytometry. Experiments were repeated for three times. ****P* < 0.001, by Student's *t*-test (mean ± standard deviation). **(D)** Iba1$^+$RUBCN$^+$ cells in lung tissue at 3d after tMCAO was quantified with immunostaining *N* = 3 in each group, **P* < 0.05, compared with PBS-treated group by one-way *ANOVA* (mean ± standard deviation), ##*P* < 0.01, compared with antibiotic-treated group by one-way ANOVA

(mean ± standard deviation). **(E)** Lung sections were subjected to immunostaining of Iba1 (green) to label macrophages, and TIM4 or TREM2 (red) to label phagocytosis associated receptors at 3d after tMCAO. **(F)** $Iba1^{+}TIM4^{+}$ and $Iba1^{+}TREM2^{+}$ cells in lung tissue at 3d after tMCAO was quantified with immunostaining. $N = 3$ in each group. **(G)** BMDM were treated with antibiotic or co-cultured with BM-MSC that stimulated with *E. Coli* or not. GFP expressing *E. Coli* were then treated to BMDM (*E. Coli* : BMDM = 20:1). Phagocytosis of the GFP expressing *E. Coli* by BMDM was assessed with immunostaining at 0.5h, 1h, 2h. Experiments were repeated for three times. **(H-I)** BMDM with *Rubcn* knock-down ($Rubcn^{KD}$) were constructed by transfection of Lentivirus carrying vector plasmid (vector) or *Rubcn*-specific shRNA. **(H)** RUBCN expression in $Rubcn^{KD}$ BMDM was assessed with western blot. **(I)** The mRNA level of the *Rubcn* was assessed with QPCR. Experiments were repeated for three times. $^{*}P < 0.05$, compared with vector-transfected group by one-way *ANOVA* (mean ± standard deviation).

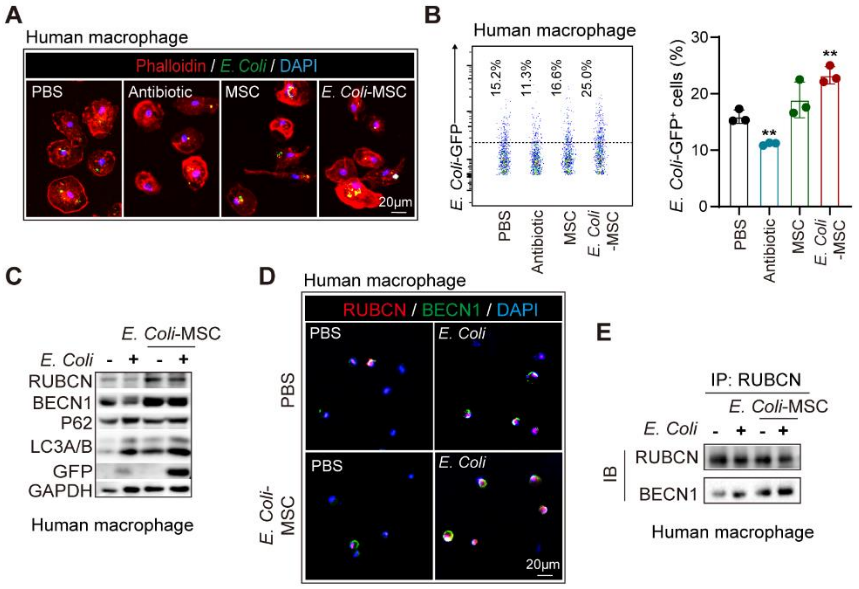

Figure S5. Bacteria-stimulated BM-MSC promotes the bactericidal function of human macrophages.

Human monocytes were isolated from healthy donors. Human macrophages were induced from the monocytes with human serum and MCSF. Human macrophages were treated with Enrofloxacin (5µg/ml, 6h) or co-cultured with BM-MSC through trans-well for overnight (pore diameter = 0.4µm, BM-MSC were stimulated with *E. Coli* for 6h or not). The macrophages were then treated with GFP-expressing *E. Coli* (*E. Coli* : macrophage = 20:1) for 1h. Phagocytic efficiency of macrophages was assessed with immunostaining **(A)** and flow cytometric analysis **(B)**. Experiments were repeated for three times. $^{**}P < 0.01$, compared with PBS group by Student's *t*-test (mean ± standard deviation). **(C-E)** Macrophages were co-cultured with *E. Coli*-stimulated BM-MSC for overnight then treated with *E. Coli* for 1h. Activation of LAP was assessed with western blot (**C**), immunostaining (**D**) and immunoprecipitation (with anti-RUBCN antibodies) and the subsequent blotting. Experiments were repeated for three times.

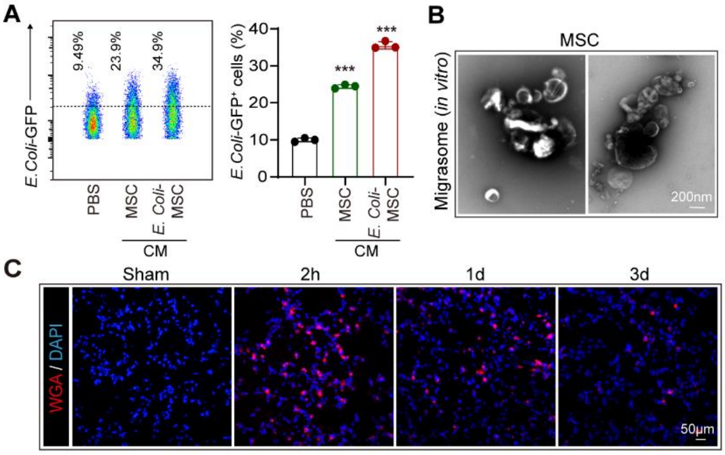

Figure S6. BM-MSC transferred to stroke recipients release migrasomes in the lung. **(A)** BMDM were pre-treated with the conditioned medium (CM) of BM-MSC overnight. GFP expressing *E. Coli* were then treated to BMDM (*E. Coli* : BMDM = 20:1). Phagocytosis of GFP expressing *E. Coli* by BMDM was assessed with flow cytometry at 1h. Experiments were repeated for three times. ***$P < 0.001$, compared with PBS-treated group by one-way *ANOVA* (mean ± standard deviation). **(B)** Transmission electron microscopy (TEM) analysis of the BM-MSC-derived migrasomes isolated *in vitro* after negative staining. Experiments were repeated for three times. **(C)** Lung sections were collected from tMCAO mice after intravenously injecting WGA-laballed BM-MSC (red, $2x10^6$ cells per mouse) for 2h, 1d and 3d to show the distribution of BM-MSC. $N = 3$ in each group.

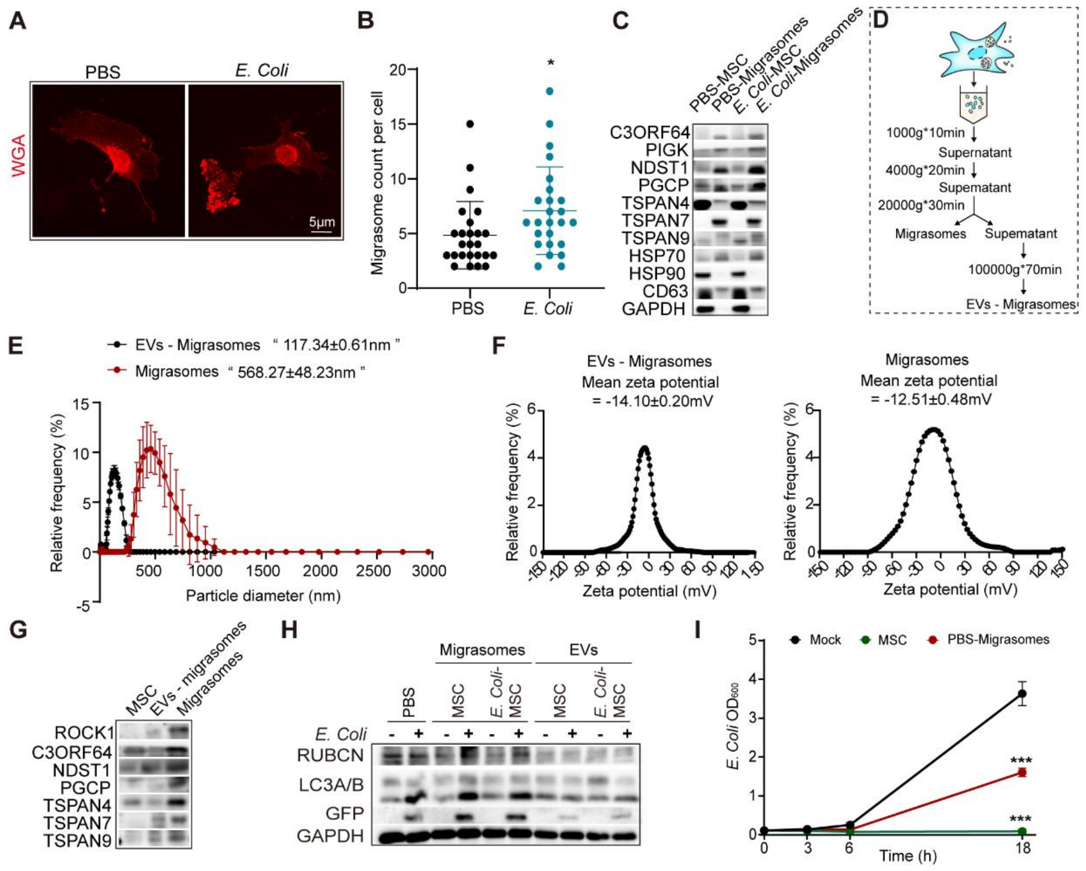

Figure S7. BM-MSC promote bacterial clearance of macrophages through releasing migrasomes.

(A-B) *E. Coli* (*E. Coli* : BM-MSC = 20:1) was applied to BM-MSC for 6h. **(A)** Staining of BM-MSC-derived migrasomes with WGA (red). **(B)** Statistics of the number of migrasomes released by BM-MSC. $N = 25$ in each group, $*P < 0.05$, compared with PBS-treated group by Student's *t*-test (mean ± standard deviation). **(C)** Expression of migrasome and exosome markers in PBS- or *E. Coli*-BM-MSC or migrasomes derived from BM-MSC were analyzed by western blot. Experiments were repeated for three times. **(D)** Schematic diagram of separation process of BM-MSC-derived migrasomes and EVs - migrasomes *in vitro*. **(E-F)** BM-MSC-derived migrasomes and EVs - migrasomes were isolated *in vitro*. Particle diameter **(E)** and Zeta potential **(F)** were measured. Experiments were repeated for three times. **(G)** Expression of migrasome markers in BM-MSC, migrasomes and EVs - migrasomes was analyzed by western blot. Experiments were repeated for three times. **(H)** BMDM were pre-treated with EVs (50μg/ml) or migrasomes (50μg/ml) that derived from BM-MSC for overnight. GFP expressing *E. Coli* were treated to BMDM for 1h (*E. Coli* : BMDM = 20:1). Expression of LAP mediators in BMDM was analyzed by western blot. Experiments were repeated for three times. **(I)** *E. Coli* growth in Luria-Bertani broth. Initial concentration of *E.*

Coli was 1x10^{7}/ml. The bacteria were cultivated in incubator at 37°C with BM-MSC coculture (*E. Coli*: BM-MSC =100:1) or PBS-migrasomes treatment (PBS-M, 50μg/ml) for indicated time period. OD600 was measured periodically. Experiments were repeated for three times. ***P < 0.001, compared with Mock group by one-way *ANOVA* (mean ± standard deviation).

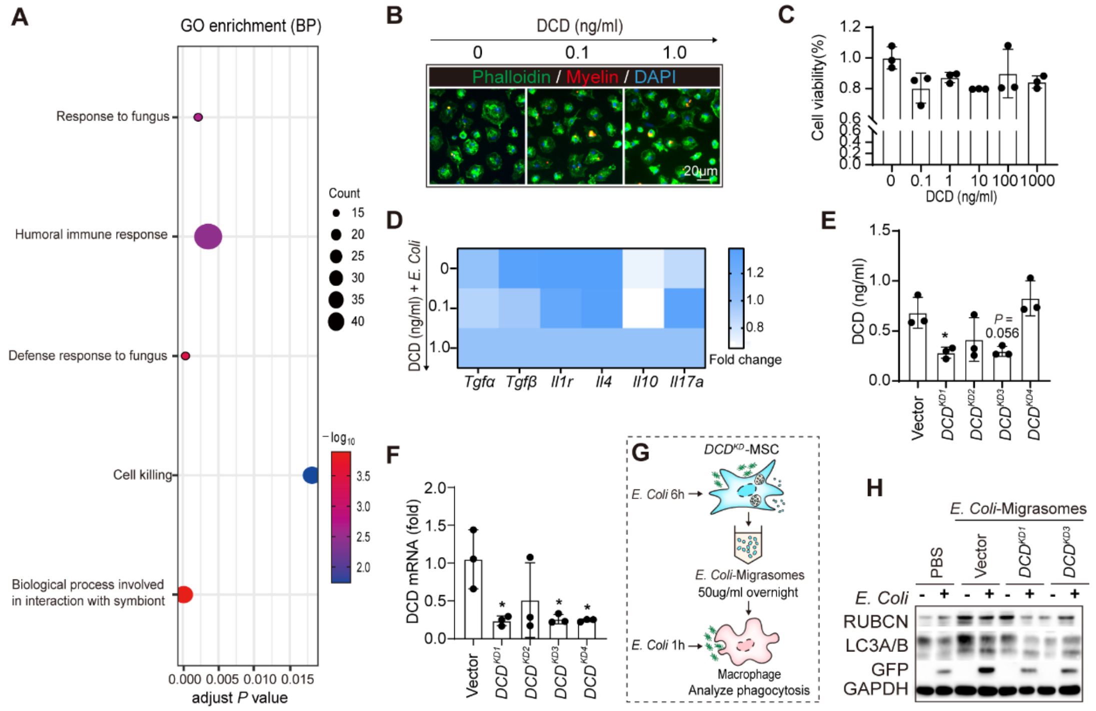

Figure S8. Effect of dermcidin (DCD) on phagocytosis and immune function of macrophages.

(A) PBS-migrasomes (PBS-M) were isolated and subjected to liquid chromatography-mass spectrometry (LC-MS) analysis. GO-BP analysis of the identified proteins in PBS-M. **(B)** BMDM were first pre-treated with DCD (0-1ng/ml, overnight), then treated with DIL-labeled myelin (100μg/ml, 1h). Phagocytic efficiency of BMDM was assessed with immunostaining. **(C)** BMDM were treated with DCD (0-1000ng/ml, overnight). Cell viability was evaluated with CCK8 colorimeter. **(D)** BMDM were pre-treated with DCD (0-1ng/ml, overnight) then simulated with *E. Coli* (*E. Coli* : BMDM= 20:1, 1h). The mRNA level of immunomodulators was assessed with QPCR. **(E)** The expression of DCD protein in *DCD* knock-down (*DCD*^{KD}) BM-MSC was assessed with ELISA. **(F)** The mRNA level of *DCD* in *DCD*^{KD} BM-MSC was assessed with QPCR. Experiments were repeated for three times. *$P < 0.05$, compared with vector-transfected group by one-way *ANOVA* (mean ± standard deviation). **(G)** *DCD* knock-down (*DCD*^{KD}) BM-MSC were constructed by transfection of Lentivirus carrying *DCD*-shRNA. *DCD*^{KD} or vector-transfected BM-MSC were stimulated with *E. Coli*. Migrasomes derived from BM-MSC were isolated and treated to BMDM (50μg/ml for overnight). Phagocytic efficiency of BMDM to GFP expressing *E. Coli* (*E. Coli* : BMDM = 20:1, 1h) was assessed. Schematic diagram of the experimental process. **(H)** Expression of LAP mediators in BMDM was analyzed by western blot. Experiments were repeated for three times.

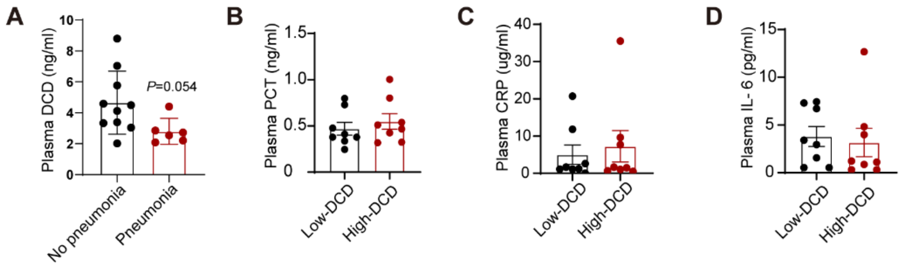

Figure S9. AIS patients with pneumonia displayed decreased plasma DCD concentration.

(A) Comparison of plasma DCD levels in AIS patients with or without pneumonia, by Student's *t*-test (mean ± standard deviation). **(B-D)** Comparison of the plasma inflammation indicators of procalcitonin (PCT, **B**), and C reactive protein (CRP, **C**) and IL-6 (**D**) levels in AIS patients with low plasma DCD concentration (DCD ≤ 3.39ng/ml, median of the cohort) or high plasma DCD concentration (DCD > 3.33ng/ml, median of the cohort).

Table S1. Demographic characteristics of the AIS patients and healthy controls.

Demographics	Healthy control (*N*=8)	AIS patient (*N*=16)	Total (*N*=24)	*P*
**Age, y, median (quartiles)**	63.5 (61.8-65.3)	64 (56.0-71.0)	63.5 (59.3-68.5)	0.8941
**Females sex, *N* (%)**	4 (50.00%)	6 (37.50%)	10 (41.67%)	0.5582
**DM, *N* (%)**	0 (0.000%)	3 (18.75%)	3 (12.50%)	0.1904
**Hypertension, *N* (%)**	5 (62.50%)	7 (43.75%)	12 (50.00%)	0.3865
**Hyperlipidemia, *N* (%)**	5 (62.50%)	2 (12.50%)	7 (29.17%)	0.0111
**CHD, *N* (%)**	0 (0.000%)	1 (6.25%)	1 (4.17%)	0.4701
**Smoke, *N* (%)**	0 (0.000%)	4 (25.00%)	4 (16.67%)	0.1213
**Alcoholism, *N* (%)**	0 (0.000%)	1 (6.25%)	1 (4.17%)	0.4701

P values were obtained by *Mann Whitney test* and *chi-square test.*

DM, diabetic mellitus. CHD, coronary heart disease.

Table S2. Primers used in the study.

Gene	Forward primer	Reverse primer
**Mus musculus**		
Rubcn	GATGGGGAGCGTCTGCTAGA	AGTCGTCTTCAAATTACCCAGC
Gapdh	CCCTTAAGAGGGATGCTGCC	TACGGCCAAATCCGTTCACA
**Homo sapiens**		
DCD	CATCCCTCTGACTTCTGTGAGCC	CTGCTGCTCCTGGGTATCATTT
GAPDH	TCGGAGTCAACGGATTTGGT	TTCCCGTTCTCAGCCTTGAC
